# Designing and comparing optimized pseudo-continuous Arterial Spin Labeling protocols for measurement of cerebral blood flow

**DOI:** 10.1016/j.neuroimage.2020.117246

**Published:** 2020-12

**Authors:** Joseph G. Woods, Michael A. Chappell, Thomas W. Okell

**Affiliations:** a Wellcome Centre for Integrative Neuroimaging, FMRIB, Nuffield Department of Clinical Neurosciences, University of Oxford, Oxford, United Kingdom; b Institute of Biomedical Engineering, Department of Engineering, University of Oxford, Oxford, United Kingdom

**Keywords:** Arterial spin labeling, ASL, Cerebral blood flow, CBF, Perfusion, Accuracy, Repeatability, Protocol optimization

## Abstract

Arterial Spin Labeling (ASL) is a non-invasive, non-contrast, perfusion imaging technique which is inherently SNR limited. It is, therefore, important to carefully design scan protocols to ensure accurate measurements. Many pseudo-continuous ASL (PCASL) protocol designs have been proposed for measuring cerebral blood flow (CBF), but it has not yet been demonstrated which design offers the most accurate and repeatable CBF measurements. In this study, a wide range of literature PCASL protocols were first optimized for CBF accuracy and then compared using Monte Carlo simulations and *in vivo* experiments. The protocols included single-delay, sequential and time-encoded multi-timepoint protocols, and several novel protocol designs, which are hybrids of time-encoded and sequential multi-timepoint protocols. It was found that several multi-timepoint protocols produced more confident, accurate, and repeatable CBF estimates than the single-delay protocol, while also generating maps of arterial transit time. Of the literature protocols, the time-encoded protocol with *T_1_*-adjusted label durations gave the most confident and accurate CBF estimates *in vivo* (16% and 40% better than single-delay), while the sequential multi-timepoint protocol was the most repeatable (20% more repeatable than single-delay). One of the novel hybrid protocols, Hybrid*_T1_*_-adj_, was found to produce the most confident, accurate and repeatable CBF estimates out of all the protocols tested in both simulations and *in vivo* (24%, 47%, and 28% more confident, accurate, and repeatable than single-delay *in vivo*). The Hybrid*_T1_*_-adj_ protocol makes use of the best aspects of both time-encoded and sequential multi-timepoint protocols and should be a useful tool for accurately and efficiently measuring CBF.

## 1 Introduction

Arterial spin labeling (ASL) MRI employs magnetically labeled arterial blood as an endogenous tracer which can be used to map cerebral blood flow (CBF) (Detre et al., 1992; Williams et al., 1992). The longitudinal magnetization of upstream arterial blood is typically labeled by inversion and, after a delay for tracer inflow (Alsop and Detre, 1996), is imaged. Images are acquired with either a single delay or multiple delays and, with the use of a control image and an appropriate signal model (Buxton et al., 1998), the local CBF can be estimated.

A consensus paper from the ISMRM Perfusion Study Group and the European ASL in Dementia consortium recommended using pseudo-continuous ASL (PCASL) labeling with a single-PLD (post labeling delay) protocol for clinical applications, due to the superior SNR of PCASL labeling and the robustness and simplicity of using a single-PLD (Alsop et al., 2015). The PLD must be set long enough to ensure complete arrival of the labeled blood across the whole brain, while being kept short enough to preserve SNR. This leads to brain regions with short arterial transit times (ATTs) having a sub-optimally long PLD, while any regions with unexpectedly long ATTs incorrectly appear hypoperfused.

Multi-timepoint protocols can be used to sample the dynamics of the tracer signal, providing greater robustness of CBF estimates to variations in ATT across brain regions and subjects as well as generating potentially useful ATT maps (MacIntosh et al., 2012). These are typically performed by sequentially changing the PLD across different measurements (multi-PLD) (Alsop and Detre, 1996) or by changing both the label duration (LD) and PLD together (Borogovac et al., 2010; Johnston et al., 2015; Zhao et al., 2015). It is often assumed that the reduction in data averaging when using multi-timepoint protocols (required when acquiring the data in a matched scan time with a single-PLD protocol) leads to a reduction in the precision of the CBF estimates (Alsop et al., 2015; Dai et al., 2017; Günther, 2007; Teeuwisse et al., 2014), which could outweigh the benefits of correcting for ATT effects. However, this neglects that the multi-timepoint data are combined during the model fitting process which could help compensate for the reduced number of averages at each timepoint.

Time-encoding of the PCASL preparation using a Hadamard encoding scheme has been proposed as a more efficient method for acquiring multi-timepoint ASL data, due to the noise averaging that occurs during the decoding process (Dai et al., 2013; Günther, 2007; Wells et al., 2010). However, this reduced noise may be counteracted by reduced ASL signal due to the shorter LDs of each sub-bolus (Guo et al., 2018). Multiple variations of the time-encoded technique have been proposed in order to improve the SNR across the different time points (Teeuwisse et al., 2014), but so far the CBF accuracy of only fixed-LD time-encoded protocols have been compared with single-PLD and sequential multi-timepoint protocols and these protocols were not explicitly optimized for CBF accuracy (Dai et al., 2013; Guo et al., 2018; Johnston et al., 2015). Therefore, the results of these comparisons may simply reflect the chosen protocol timings rather than the ultimate accuracy of each technique.

We recently demonstrated that a sequential multi-PLD PCASL protocol can be objectively optimized to maintain higher CBF accuracy across a wider range of ATTs than a single-PLD or evenly spaced multi-PLD protocol (Woods et al., 2019). This was due to an improved balance between early sampling of the tracer kinetics (which has higher SNR and benefits short ATT brain territories) with late sampling (which has lower SNR and benefits long ATT territories). So far, this optimization framework has only been applied to sequential multi-PLD PCASL protocols with a fixed and unoptimized label duration.

**Fig. 1:**
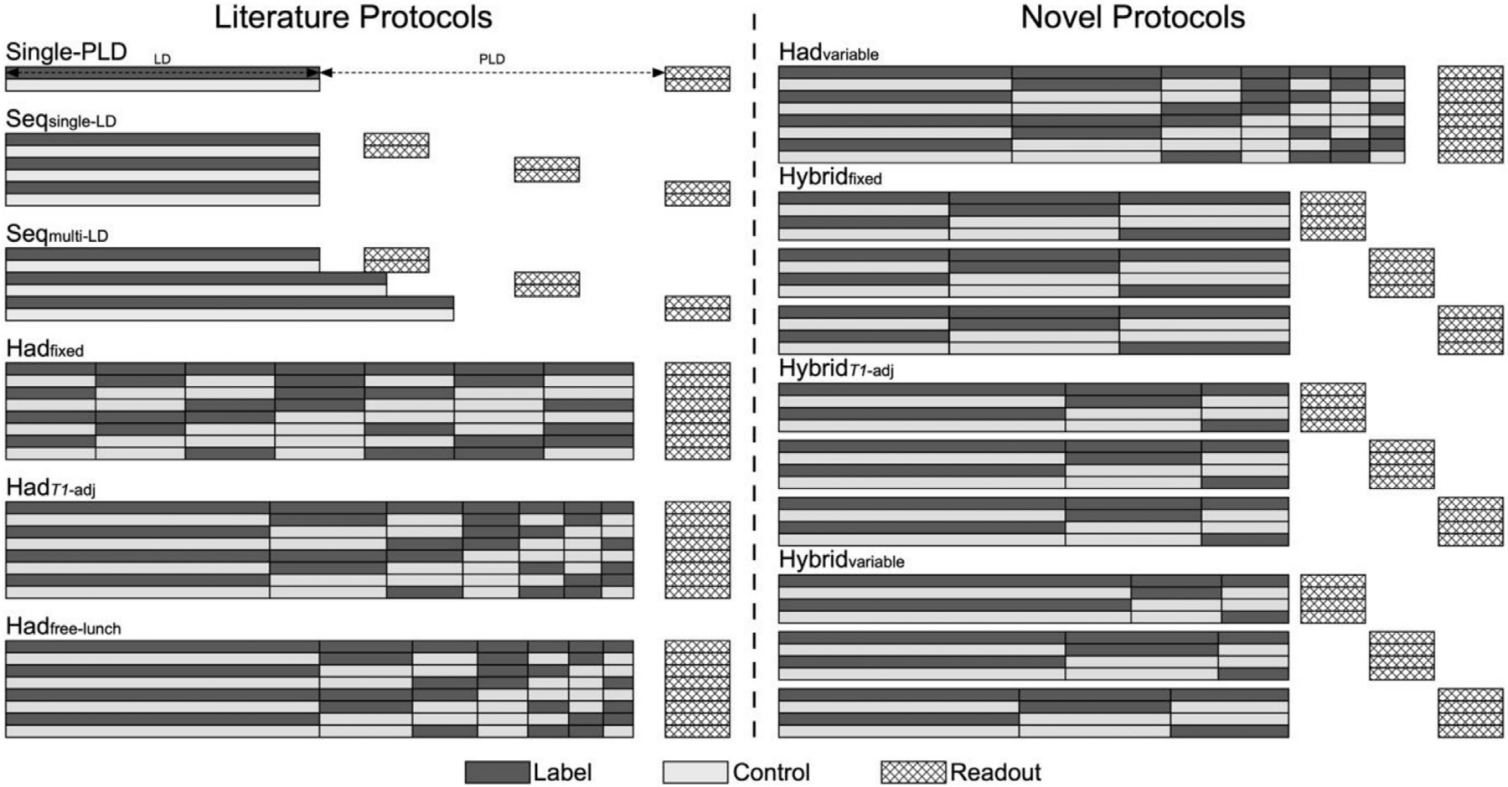
Example timing schematics of the PCASL label/control protocols used in this work. The label duration, post labeling delays, number of label/control pairs, and the size of the time-encoding matrices were optimized for the multi-timepoint protocols; see Section 3.1 for details.

In this study, we aimed to establish which PCASL approach can achieve the most accurate CBF measurements. We did this by comparing the CBF accuracy of a single-PLD protocol, a wide range of multi-timepoint PCASL protocol designs from the literature, and several novel hybrid protocol designs which are introduced in this study (Fig. 1). We first applied a previously developed optimization framework (Woods et al., 2019) to the multi-timepoint protocol timings to ensure each would optimally estimate CBF across an expected range of ATTs for healthy gray matter (GM) given the design constraints of each protocol. The CBF accuracy of these optimized protocols were then compared using Monte Carlo (MC) simulations, with a subset of protocols being compared *in vivo*. This study builds on work previously presented in abstract form (Woods et al., 2018a, 2018b).

## 2 Theory

### 2.1 Literature protocol designs

The range of protocol designs investigated in this work are shown in Fig. 1. The single-PLD and sequential multi-PLD, with a fixed LD, (Seq_single-LD_) protocol designs have been widely used in the literature to estimate CBF only or both CBF and ATT, respectively (Alsop et al., 2015; Alsop and Detre, 1996; Buxton et al., 1998; Dai et al., 2017; Gonzalez-At et al., 2000; Okell et al., 2013). Borogovac et al. (2010) suggested the use of multiple sequential LDs with a fixed PLD as a more SNR-efficient method for estimating CBF and ATT than fixed-LD multi-PLD methods, though this hypothesis was not tested. Johnston et al. (2015) later demonstrated the use of both varying LDs and PLDs (referred to here as Seq_multi-LD_) to estimate CBF and ATT, but this implementation did not use inversion pulses for background suppression (BGS), instead relying only on pre-saturation to facilitate *T_1_* estimation from the ASL data, which may have affected the resulting CBF accuracy. In this study, we investigated both Seq_single-LD_ (a single fixed LD with *N* PLDs) and Seq_multi-LD_ (*N* LDs paired with *N* PLDs) protocols.

Günther (2007) introduced time-encoded PCASL as an efficient method for generating multi-timepoint ASL data. The PCASL pulse train is split into *M* sub-boluses which vary between label and control conditions within each TR according to a predesigned encoding matrix (a Hadamard matrix being the most efficient encoding). The acquired data is then decoded, generating *M* perfusion weighted images which reflect the effective LD and PLD of each sub-bolus. For a Hadamard encoding of size (M+1)×M, the averaging effect of decoding results in a factor of $\sqrt{(M+1)/2}$decrease in noise standard deviation (SD) (assuming additive white Gaussian noise) and a reduction in scan time by a factor of (2·M)/(M+1)compared to a matched timing sequential control - tag experiment (Dai et al., 2013).

The original time-encoded protocol used a fixed LD for all sub-boluses (Had_fixed_). Several variations were introduced by (Teeuwisse et al., 2014), including the free-lunch (Had_free-lunch_) and *T_1_*-adjusted (Had*_T1_*_-adj_) protocols. In the *T_1_*-adjusted protocol, the encoded LDs are set such that the total ASL signal originating from each sub-bolus is equal at the time of acquisition, thus accounting for the increased *T_1_* decay experienced by earlier sub-boluses and so maintaining an approximately constant level of SNR after complete bolus arrival. The free-lunch protocol uses the same long LD and PLD for the first encoded sub-bolus as a typical single-PLD protocol, with the remaining sub-boluses filling this long PLD. After decoding, similar data to the single-PLD experiment is generated from the first sub-bolus, with the remaining sub-boluses generating extra temporal data without an increase in scan time. Fig. 1 shows Had_free-lunch_ with the remaining sub-boluses having *T_1_*-adjusted LDs, but any scheme may be used.

### 2.2 Hybrid protocol designs

Here, we introduce a novel protocol design which is a hybrid of the time-encoded and sequential protocols. Rather than using a fixed final PLD after the time-encoded preparation and acquiring multiple averages, there are *N* final PLDs which sequentially vary for each repeat of the same encoding matrix, allowing increased flexibility of the decoded timepoints. This results in *N* · *M* decoded timepoints (*N* final PLDs, *M* time-encoded sub-boluses). This design can trade-off the superior noise averaging of the time-encoding methods (larger encoding matrices result in more signal averaging) and the increased signal accumulation from longer LDs (typically achievable with smaller encoding matrices), whilst maintaining flexible sampling of timepoints (achieved by varying the final PLD). We investigated the use of both fixed (Hybrid_fixed_) and *T_1_*-adjusted (Hybrid*_T1_*_-adj_) time-encoded LDs with this protocol design.

### 2.3 Time-encoded and hybrid designs with variable-LDs

The time-encoded and hybrid protocols do not have to be restricted to the designs discussed above, i.e. fixed and *T_1_*-adjusted LDs. It is possible for the individual encoded LDs and final PLDs to be chosen arbitrarily. As an extension to the comparison of the protocols detailed above, we tested whether there is a more optimal time-encoded LD pattern than the existing literature designs by optimizing a time-encoded protocol and a hybrid protocol where each LD in the encoding matrix could be adjusted separately, rather than according to a predefined pattern. To increase the flexibility of the hybrid protocol even further, each of the *N* final PLDs was associated with a separate encoding matrix of *M* LDs, rather than repeating the same encoding matrix timings for each of the PLDs, leading to *N* · *M* decoded timepoints with *N* · *M* separate LD and PLD pairs. These protocols are referred to as Had_variable_ and Hybrid_variable_.

## 3 Material and methods

All optimizations, simulations and analysis, except CBF and ATT estimation, were performed using MATLAB (The MathWorks, Natick, MA).

### 3.1 Protocol optimization

The multi-timepoint protocols described above were optimized for CBF accuracy, while treating ATT as a potentially confounding parameter. This was achieved using a recently developed framework using the L-optimal cost function with non-zero weighting on CBF accuracy and zero weighting on ATT accuracy (Woods et al., 2019). The original implementation of the optimization algorithm iterated through each of the *N* PLDs of a multi-PLD protocol, and for each, performed a grid search for the PLD value which minimized the mean Cramér-Rao Lower Bound (CRLB) variance across ATTs, taking into account the number of averages realizable in a given scan time. The principal of the optimization for each protocol considered in this work was the same, with the addition of also optimizing the LDs, but due to the different sizes of the timing parameter spaces, the implementation was adapted in each case, as described in Supporting information text 1. For each protocol, the number of effective PLDs, *N_T_*, was optimized by running the optimization for a range of *N_T_* and selecting the protocol with the minimum cost. *N_T_* was constrained to ≤15 to ensure that either multiple averages at each timepoint can be performed or a segmented readout can be used with these protocols. The single-PLD protocol was not optimized with this framework; instead, the LD and PLD were set to 1.8 s and 2 s, respectively, which are recommended for clinical populations (Alsop et al., 2015).

The optimization used a uniform ATT prior probability distribution with a representative GM range of 0.5 - 2 s for healthy volunteers (Alsop et al., 2015; Dai et al., 2017; Guo et al., 2018; Woods et al., 2019), sampled at 1 ms increments, with a 0.3 s linearly decreasing weighting beyond either end of the range to reduce edge effects. Since the optimization does not depend on CBF (Woods et al., 2019), a CBF point prior of 50 mL/100 g/min was used. The LD update grid searches were restricted to 0.1 *s* ≤ LD ≤ 1.8 s with 25 ms increments, ensuring the minimum LD was greater than 100 ms, as suggested by Teeuwisse et al. (2014), with the longest LD matching the recommended single-PLD LD of 1.8 s (Alsop et al., 2015). In the case of the time-encoded protocols, these LD restrictions were placed on the sub-bolus durations, with there being no constraint placed on the total duration of the time-encoded preparation. The PLD update grid was 0.075 *s* ≤ PLD ≤ 2.3 s with 25 ms increments, since PLDs longer than the longest ATT are not selected by the optimization algorithm (Woods et al., 2019). Other settings included: single-shot readout with 638 ms of non-ASL time per TR (presaturation and readout); variable minimum TR (Wang et al., 2013) (where the TR is minimized for each timepoint); 5 min scan duration. The CRLB was calculated using the standard CASL kinetic model from (Buxton et al., 1998), using the parameters in Table 1, and assumed additive white Gaussian noise, as described in (Woods et al., 2019). The noise magnitude was calculated from preliminary *in vivo* data (noise SD of label and control data = $1.3\;\times 10^{-3}$relative to *M_0_*).

**Table 1:** Model and sequence parameters used in the optimizations, Monte Carlo simulations and *in vivo* experiments.

**Parameter**	**Value**
***Model***	
*T_1_* of blood (*T_1b_*)	1.65 s (Lu et al., 2004)
*T_1_* of tissue (*T_1t_*)	1.445 s (Lin et al., 2001)
Labeling efficiency (α)	0.85 (Dai et al., 2008)
Brain/blood water partition coefficient (λ)	0.9 mL/g (Herscovitch and Raichle, 1985)
***Sequence***	
RF labeling pulse duration	500 µs duration (Gaussian)
RF labeling pulse interval	1 ms
RF labeling flip angle	20°
Mean labeling gradient	0.8 mT/m
Gradient during labeling pulses	6 mT/m
***Analysis***	
CBF prior	0 ± 10^6^ mL/100 g/min
ATT prior	1.3 ± 10^6^ s

The MATLAB-based optimization code used for this study is available at https://github.com/JosephGWoods/ComparisonPCASLProtocolOptimisation and an open-source python-based GUI and command line tool is available at https://github.com/ibme-qubic/oxasl_optpcasl.

### 3.2 Monte Carlo simulations

Monte Carlo simulations were performed to evaluate the optimized protocols under ideal conditions where the ground truth CBF and ATT are known. Simulated data were generated for each protocol using the standard CASL kinetic model (Buxton et al., 1998) with the parameters in Table 1 for ATTs between 0.5 - 2 s at 0.01 s increments. White Gaussian noise was added to 2000 replicas of the label and control (or encoded) data at each ATT sample, using the same noise magnitude as the protocol optimizations above. The noisy data at each timepoint was then subtracted or decoded according to the encoding scheme for each protocol. The data were then fit, and the estimates compared, as described below.

### 3.3 *In vivo* experiments

#### 3.3.1 Acquisition

To investigate the relative performance of the protocols given in Table 2 *in vivo*, 10 healthy volunteers (5 female, mean age 30.7, range 24 - 40) were recruited and scanned with written informed consent under a technical development protocol, agreed with local ethics and institutional committees, on a 3T Prisma system (Siemens Healthcare, Erlangen, Germany) with a 32-channel receive-only head coil. The Had_variable_ and Hybrid_variable_ protocols were not compared *in vivo* since they only led to marginal improvements in CBF accuracy during simulation (see Results, Section 4.9). All scanning occurred during a single session for each subject (total scan duration ~50 min). Volunteers were asked to lie still and stay awake throughout the scan. A nature documentary was shown to help maintain alertness.

**Table 2:** The optimized protocol timings for the protocols compared *in vivo* and in simulation. For the time-encoded (Had) and hybrid protocols, the LDs are given in chronological order and the number of LDs defines the size of the Hadamard encoding matrix used. For the Hybrid_variable_ protocol, each PLD is associated with the LDs on the same row, the number of columns defining the size of the Hadamard encoding matrix. *N_T_* is the number of effective PLDs, *N_Ave_* is the number of averages, and *N_Acq_* is the number of acquired volumes for each scan.

Protocol	Label durations (ms)	Post label delays (ms)	*N_T_*	*N_Ave_*	*N_Acq_*	Scan duration (min)
***Simulation and in vivo comparison***						
***Single-PLD***	1800	2000	1	34	68	5:02
***Seq_single-LD_***	1800	175, 1050, 1425, 1725, 2075, 2200, 2300, 2300, 2300	7	4	72	5:00
***Had_fixed_***	550, 550, 550, 550, 550, 550, 550	100	7	8	64	4:54
***Had_T1-adj_***	1150, 675, 475, 375, 300, 250, 225	75	7	9	72	5:00
***Had_free-lunch_***	1800, 625, 450, 350, 300, 250, 225	125	7	8	64	5:05
***Hybrid_fixed_***	1275, 1275, 1275	75, 150, 600, 850, 1000	15	3	60	5:00
***Hybrid_T1-adj_***	1800, 850, 550	200, 650, 900, 900	12	4	64	4:48
***Simulation comparison only***						
***Had_variable_***	1725, 750 650, 375, 150, 150, 125	100	7	8	64	4:58
***Hybrid_variable_***	1800, 1050, 775	100	12	4	64	4:55
	1800, 1225, 550	525				
	1800, 850, 750	575				
	1800, 800, 800	700				

The scan protocol included a 3-plane localizer and a 3D single-slab TOF angiography sequence used to position the PCASL labeling plane. A 3D *T_1_*-weighted MPRAGE sequence (1.5 × 1.5 × 1.5 mm^3^) was acquired for generating the brain and gray matter (GM) masks. Four calibration images were acquired with the same readout module as the PCASL data (see below) but with alternating in-plane phase encode direction to correct off-resonance distortions. Finally, the ASL scans were acquired in a pseudo-randomly permuted order for each subject to reduce the impact of physiological drift.

ASL imaging parameters were: single-shot 3D gradient and spin-echo (GRASE) readout (Feinberg and Oshio, 1991; Günther et al., 2005), TE 28.5 ms, variable minimum TR, excitation flip-angle 90°, refocusing flip-angle 120° (He et al., 2018; von Samson-Himmelstjerna et al., 2016), FOV 230 × 168 × 100 mm^3^, matrix 64 × 46 × 20, 20 acquired partitions, no parallel imaging acceleration, no slice-oversampling, centric partition ordering, bandwidth 2298 Hz/px, total readout duration 583 ms, spectrally-selective fat saturation. The imaging slab was placed in the transverse plane with the superior edge flush with the top of the brain. The excitation and refocusing pulse widths were 110 mm and 150 mm, respectively, to maximize the signal level within the nominal slab. Outer-volume suppression (OVS), using a cosine-modulated water suppression enhanced through *T_1_* effects (WET) module (Golay et al., 2005; Ogg et al., 1994), was used to improve the slab profile, similar to (Günther et al., 2005). Readout, phase-encode, and 3D encode directions were anterior-posterior, right-left, and feet-head, respectively.

PCASL labeling was achieved using the parameters in Table 1 with the labeling plane positioned in the transverse plane bisecting the V3 section of the vertebral arteries (Okell et al., 2013). Background suppression was performed with a slab-selective WET presaturation module (Golay et al., 2005; Ogg et al., 1994) immediately before the start of labeling and two optimally timed slab-selective C-shaped FOCI pulses (µ = 1.5, β = 1349 s^−1^, *A_max_* = 20, duration 10.24 ms) (Ordidge et al., 1996; Payne and Leach, 1997). The presaturation and inversion slabs were parallel to the labeling plane and covered the entire brain, with the inferior edge at the center of the labeling plane. For each protocol, the inversion pulses were timed to null two *T_1_* values (700 ms and 1400 ms) 100 ms before excitation using the formula in (Günther et al., 2005). The inversion pulses were interleaved with the PCASL labeling when the optimal inversion times occurred during the labeling period, as in (Dai et al., 2012, 2010), leading to more uniform BGS across a range of timings (Supporting Information Figure S1).

The calibration images were acquired using presaturation followed by a 10 s delay to allow controlled and near-complete magnetization recovery before the 3D-GRASE readout.

#### 3.3.2 Preprocessing

Preprocessing of the *in vivo* data was performed using tools from the FSL toolbox (Jenkinson et al., 2012). The ASL data were motion-corrected and registered to the mean calibration data with rigid-body registration using FLIRT (Jenkinson, 2002; Jenkinson and Smith, 2001), before correcting for susceptibility induced off-resonance geometric distortions using the calibration images with TOPUP (Andersson et al., 2003). Brain and GM masks were generated from the structural data using BET (Smith, 2002) and FAST (Zhang et al., 2001). These were transformed to ASL space after image registration (Greve and Fischl, 2009) and had thresholds applied (brain mask 90%, GM mask 50% tissue partial volume).

The edges of the brain-masked calibration image were eroded before being expanded using a mean filter and brain masked again to remove low-intensity voxels at the edge of the brain which can lead to erroneously high CBF values during the voxelwise calibration step. It was then smoothed (Gaussian kernel, σ = 2.5 mm) to improve SNR, as recommended (Alsop et al., 2015).

The perfusion-weighted images were generated by pairwise subtracting or decoding the preprocessed ASL images. They were then calibrated prior to fitting to account for scaling factors by voxelwise dividing by the smoothed calibration image and the labeling efficiency and multiplying by the blood–brain partition coefficient (Table 1).

#### 3.3.3 Model fitting

CBF and ATT were estimated identically for the simulated data and *in vivo* data using the variational Bayesian inference algorithm, BASIL (Chappell et al., 2009). In each voxel, this approach not only provides estimates of CBF and ATT but also uncertainty estimates in the form of the standard deviation of the marginal posterior distributions. The standard CASL kinetic model (Buxton et al., 1998) was used with the parameters in Table 1. Fitting was initialized with a coarse grid search for robustness (bounded by 0 ≤ CBF ≤ 200 mL/100 g/min and 0 ≤ ATT ≤ 2.5 s, sampled every 1 mL/100 g/min and 0.01 s). The BASIL fitting priors were noninformative to minimize bias in the resulting parameter estimates. Neither spatial regularization nor partial volume correction were used. Negative CBF and ATT estimates were set equal to zero. The single-PLD data was only fit for CBF with the ATT fixed at 1.3 s; this value was found to minimize the theoretical CBF bias across the ATT range 0.5–2 s. The data were not averaged before fitting to allow the algorithm to estimate the noise in the data.

#### 3.3.4 Ground truth estimates for accuracy comparison

*In vivo* ground truth CBF and ATT estimates were generated by fitting the combined data from all protocols (~35 min of data), similar to (Woods et al., 2019). To account for the different noise levels between protocols, BASIL was given 3 noise magnitudes to estimate in an approach similar to weighted NLLS fitting (Chappell et al., 2009). Three noise magnitudes were used because there were 3 categories of data with similar noise magnitudes after decoding: the non-time-encoded data (single-PLD and sequential protocols), the 8 × 7 Hadamard encoded protocols, and the 4 × 3 Hadamard encoded hybrid protocols (see Results, Section 4.1). To investigate whether these ground truth estimates were biased towards certain protocols and whether modelling the 3 noise magnitudes is beneficial, ground truth estimates for the MC simulation data were identically generated with either 1 or 3 noise magnitudes.

#### 3.3.5 Comparison metrics

The CBF estimates of each protocol were compared in three different ways for both simulation and *in vivo* data: (1) the marginal posterior probability distribution standard deviations (SDs) output by BASIL were used as a measure of uncertainty in the CBF estimates (Chappell et al., 2009), and are sensitive to how well the kinetic model fits the data; (2) the root-mean-squared-error (RMSE) relative to the ground truth estimates were used as a measure of accuracy, incorporating both systematic bias and noise contributions, similar to (Woods et al., 2019); and (3) the test-retest RMSE for each protocol was calculated by splitting the data into two 2.5 min data sets and separately fitting each half, giving a measure of within-session repeatability, which is independent of any ground truth estimates or uncertainties derived from the fitting process. Note, for (3) the Had*_T1_*_-adj_ data were split into the first 4 and last 5 averages while the Hybrid_fixed_ errors could not be calculated because there were only 3 averages (see Table 2).

#### 3.3.6 *In vivo* data exclusion and standard errors

Only voxels within the GM masks were used in the analysis. To eliminate poorly fit ground truth data from the analysis, voxels with ground truth posterior SDs more than 3 times the inter-quartile range above the 75th percentile for either CBF or ATT were excluded (Tukey, 1977). This resulted in upper bounds on the ground truth posterior SDs of 2.9 mL/100 g/min and 0.061 s for CBF and ATT, respectively. Voxels were also excluded if the posterior SDs for any individual protocol were >500 mL/100 g/min or >50 s, which would suggest an extremely poor fit, perhaps arising from motion or other artefacts, and could bias the resulting comparison. This extremely poor fit criteria were also used in the MC simulation analysis.

The comparison metrics were calculated on a voxelwise and subjectwise basis. Standard errors for the voxelwise metrics were calculated by bootstrap sampling (Efron, 1979) across the 10 subjects using 1000 samples, where the relevant statistical measure (mean SD, RMSE, or test-retest RMSE) was performed on each bootstrap sample. Each sample is a selection of 10 randomly chosen subjects, selected with replacement, meaning a given sample could contain multiple copies of the same subject's data. The SDs generated from these bootstrap distributions reflect the variability in the voxelwise metrics due to the sampled subjects. This approach gives a more conservative standard error than would be calculated from the combined voxelwise data across all subjects due to the large number of voxels.

## 4 Results

### 4.1 Optimized protocols

The optimized timings for each protocol are shown in Table 2. The increasing density of samples at later timepoints is characteristic of protocols optimized for CBF accuracy (Woods et al., 2019). When the label duration was allowed to vary between measurements in the sequential design (Seq_multi-LD_), the optimal protocol (Supporting Information Table S1) was very similar to that of the fixed label duration protocol (Seq_single-LD_, Table 2), giving only a marginal improvement in predicted CBF errors. Seq_multi-LD_ was therefore not used in further comparisons.

For the time-encoded protocols, a 4 × 3 encoding came out as optimal when each sub-bolus had a fixed duration (Had_fixed_, see Supporting Information Table S1 and Supporting Information Figure S2). However, due to the more common use of the 8 × 7 encoding, only the 8 × 7 protocol was used in further comparisons. For the free-lunch approach (Had_free-lunch_), the optimized protocol used an 8 × 7 encoding with the remaining sub-boluses having *T_1_*-adjusted durations. The novel hybrid protocols made use of fewer, but longer sub-boluses with a 4 × 3 encoding matrix combined with multiple final PLDs to allow the sampling of many timepoints.

When complete freedom in the sub-bolus durations was allowed for the time-encoded and hybrid protocols (Had_variable_ and Hybrid_variable_), only marginal improvements in predicted CBF accuracy were achieved. These approaches were therefore only used in simulation and their results reported separately in Section 4.9.

### 4.2 *In vivo* CBF and ATT maps

**Fig. 2:**
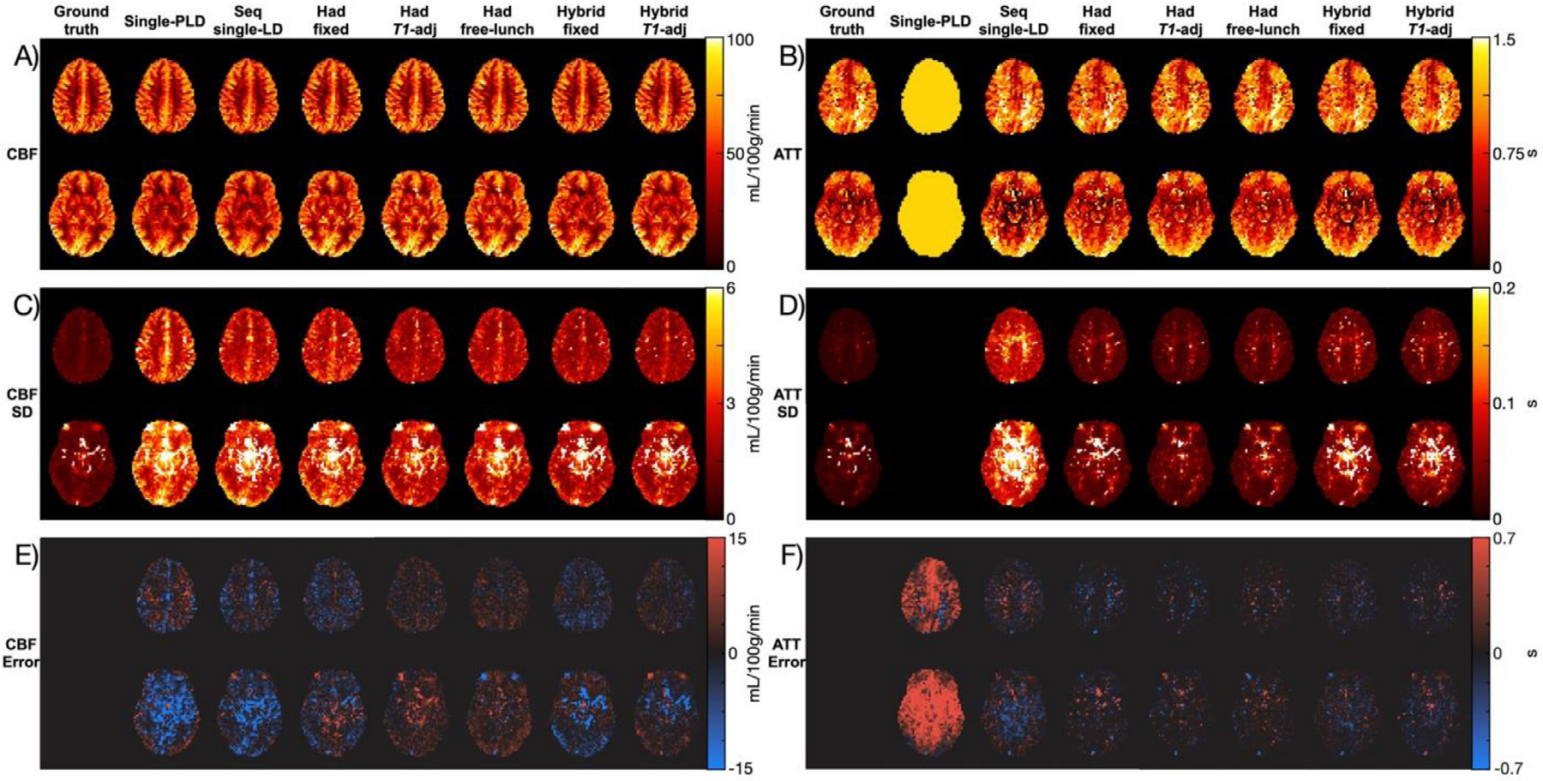
The CBF (A, C, E) and ATT (B, D, F) mean estimates (A, B), uncertainties (C, D), and errors relative to the ground truth estimates (E, F). The uncertainties are expressed as the standard deviation of the marginal posterior probability distribution output by BASIL. The error maps are shown as $estimates_{protocol}-estimates_{ground\;truth}$. Two slices from a representative subject are shown. The color maps are designed for perceptually uniformity, developed by (Kovesi, 2015).

Fig. 2 shows the spatial maps of the CBF and ATT estimates, their uncertainties (expressed as the SD of the posterior distributions), and the errors relative to the ground truth estimates for each tested protocol for a single representative subject. The CBF and ATT maps are shown for all subjects in Supporting Information Figure S3 and Supporting Information Figure S4. There is good agreement in the broad spatial variations of both CBF and ATT between the protocols, demonstrating the overall consistency of the estimates. However, the error maps highlight the variation between protocols in over/under-estimating CBF and ATT. Particularly evident, is the effect that the assumed single-PLD ATT had on the single-PLD CBF errors: regions where the assumed ATT was an overestimate led to the CBF being underestimated, relative to the ground truth estimates. It is also apparent that there was a greater CBF uncertainty associated with the single-PLD protocol than many of the multi-timepoint protocols across most of the brain.

High uncertainties in the lower slice of the CBF and ATT SD maps can be seen in regions consistent with the known location of large arteries in all of the protocols. Due to the presence of these elevated SDs in the single-PLD data, which has a long PLD of 2 s, it was assumed to be largely caused by signal dephasing due to pulsatile flow during the GRASE readout, rather than macrovascular ASL signal. However, these large arteries may contain residual ASL signal for the protocols with short PLDs. High uncertainties can also be seen in the sagittal sinus and from eye motion. These voxels were not included in the quantitative comparisons (see Section 4.3).

### 4.3 *In vivo* data selection

There were a total of 79,211 voxels in the GM masks across all 10 subjects. Of these, 6.2% were excluded due to poor ground truth CBF and ATT fits (posterior SDs >2.9 mL/100 g/min or >0.061 s) and a further 4.1% were excluded because there were extremely poor fits in one or more of the individual scans (posterior SDs >500 mL/100 g/min or >50 s). Supporting Information Figure S5 shows, for a single subject, that the excluded voxels are mostly located where one would expect large arteries to be.

For the included voxels, the mean gray matter CBF estimates were not significantly different across protocols on the subject level (Wilcoxon signed rank test, Bonferroni correction for 21 comparisons, *p* ≥ 0.05), averaging at 57.16 ± 0.52 mL/100 g/min (mean ± SD across protocols). Of the included voxels, 90% of the ground truth ATTs lay between 0.5 - 1.51 s (5th–95th percentiles, median = 0.97 s).

### 4.4 Variation in CBF uncertainty with ATT

**Fig. 3:**
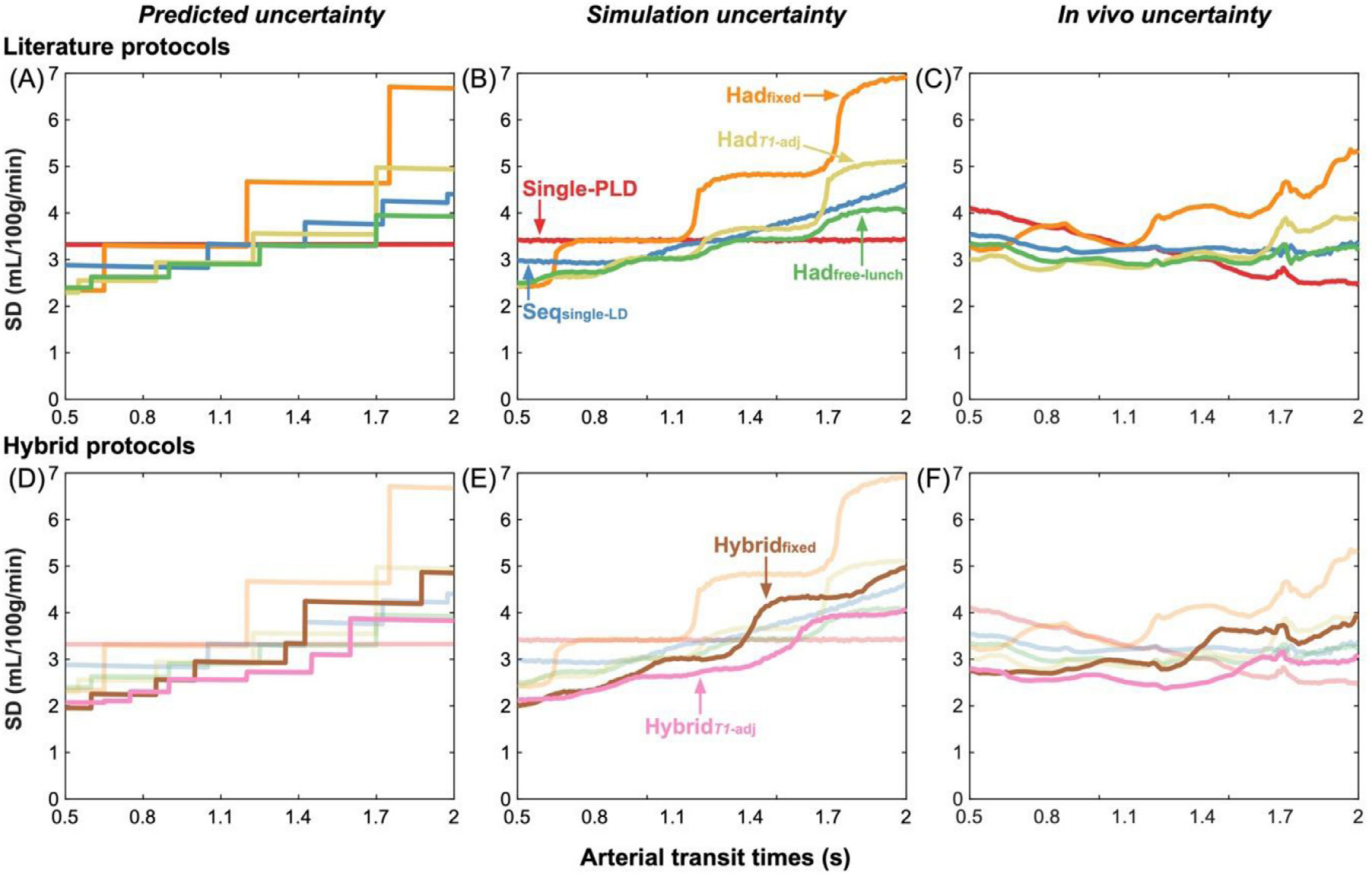
The uncertainty in CBF estimates predicted by theory (Cramér–Rao lower bound SDs) (A,D), derived from simulations (posterior SDs) (B,E), and from *in vivo* data (posterior SDs) (C,F) shown as a function of ATT. Literature protocols are compared in the top row. The proposed hybrid protocols are overlaid on faded plots of the literature curves on the bottom row to aid visualisation. For the simulation and *in vivo* results, the median SD at each ATT is plotted. A sliding window was used to plot the *in vivo* data with window size 0.1 s and step size 0.01 s.

The predicted CBF uncertainties (the CRLB SDs) for the literature protocols and novel hybrid protocols are shown in Fig. 3(A, D) as a function of ATT for a fixed CBF of 50 mL/100 g/min. The single-PLD CBF uncertainties were flat across the ATT range because they depend on the assumed fixed ATT (1.3 s), not the true ATT, and the noise magnitude, which is assumed constant across all true ATTs (see Discussion Section 5.3.2). The sharp changes in uncertainties across ATTs for the multi-timepoint protocols are where ATT=PLD or ATT=LD+PLD for one or more of the LD/PLD pairs. As the ATT increases, these discontinuities represent the transition of a data point to either no longer sampling the inflow section of the kinetic model (LD+PLD<ATT) or moving from the tracer decay portion of the model (ATT<PLD) to the inflow portion (ATT<LD+PLD<LD+ATT). Both cases result in an increase in the CBF uncertainty due the sharp increase in sensitivity to ATT in the model at these points (Woods et al., 2019).

Of the literature protocols, the time-encoded free-lunch protocol (Had_free-lunch_) maintained the lowest uncertainties across most of the ATT range. The time-encoded protocol with *T_1_*-adjusted sub-bolus durations (Had*_T1_*_-adj_) performed similarly to the free-lunch approach at short ATTs, reflecting the similarity in the timings of their last 6 time-encoded sub-boluses, but had much larger uncertainties at ATT > 1.7. The sequential multi-PLD protocol (Seq_single-LD_) maintained similar uncertainties across the ATT range to the best time-encoded protocols. However, time-encoding with a fixed sub-bolus duration (Had_fixed_) performed much worse than the other protocols across most of the ATT range. The multi-timepoint protocols all had reduced uncertainties at short ATTs compared to the single-PLD protocol but were worse at longer ATTs.

Both hybrid protocols achieved lower predicted CBF uncertainties at almost all ATTs relative to their non-hybrid analogues. The hybrid approach with *T_1_*-adjusted sub-bolus durations (Hybrid*_T1_*_-adj_) also maintained lower uncertainties than all other multi-timepoint protocols at almost all ATTs and had lower uncertainties than the single-PLD protocol for most of the ATT distribution.

The median CBF uncertainties from the Monte Carlo simulations (the marginal posterior probability distribution SDs from the Bayesian fitting) are shown in Fig. 3(B, E) and follow the trends of the predicted uncertainties extremely closely, validating the expected performance of each protocol under ideal conditions. The CBF uncertainty discontinuities are visible but are more gradual due to the blurring effect of noise on ATT estimation.

The *in vivo* median CBF posterior uncertainties (Fig. 3(C, F)) exhibit similar relative performance for each protocol, but there is a general decrease in the uncertainties at longer ATTs for all protocols compared to the predicted and simulation CBF uncertainties. This is thought to be due to the correlation between ATT and the temporal SNR (see Discussion, Section 5.6). Similar jumps in the uncertainties can be seen, especially for the Had_fixed_ protocol, however, these are somewhat smoother than in the simulations, possibly due to differences in the noise (see Section 5.6) and dispersion of the bolus *in vivo* (Chappell et al., 2013).

### 4.5 Comparison of protocols: uncertainty

**Fig. 4:**
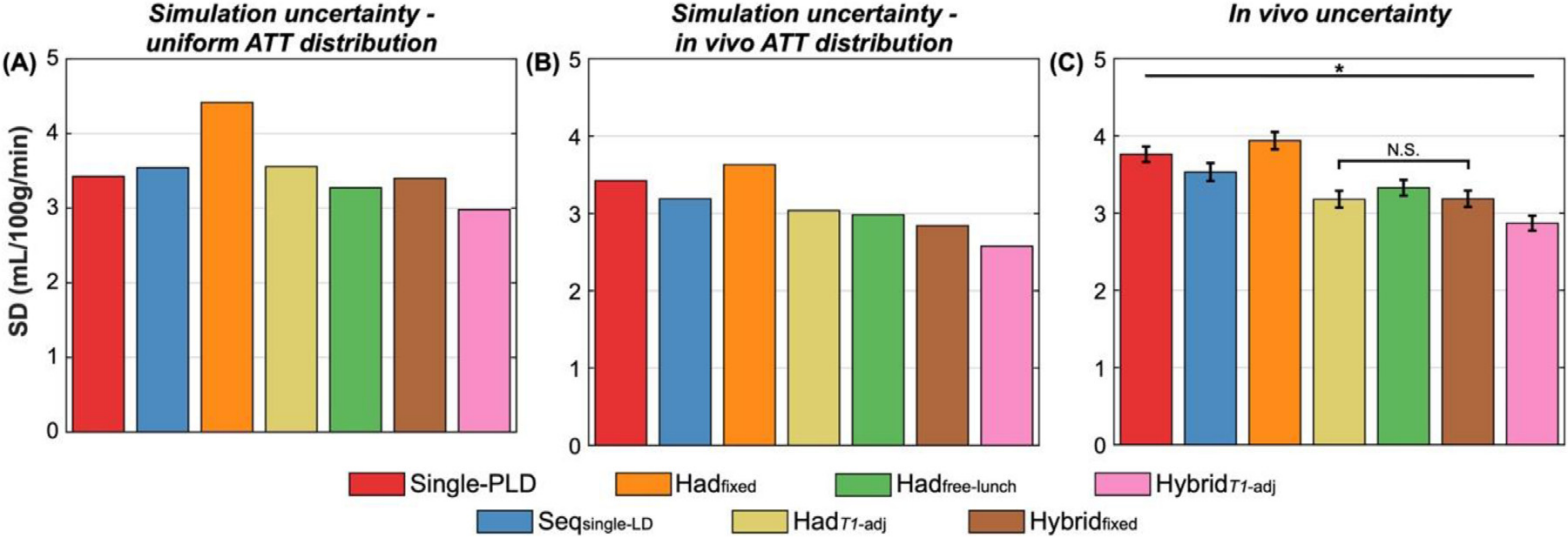
The uncertainty in CBF estimates (mean posterior SDs) derived from MC simulations (A, B) and *in vivo* data (C) across all voxels. (A) shows the simulation results for the uniform ATT distribution, while (B) shows the simulation results weighted by the measured *in vivo* ground truth ATT distribution. *In vivo*, the means and standard errors of the bootstrap distributions are shown (see Methods section 3.7). All differences were significant except for *Had*_T1-adj_ vs Hybrid_fixed_*in vivo* (two-sided paired Wilcoxon signed-rank test, Bonferroni correction for 21 comparisons, *p* < 0.05).

The mean MC simulation and *in vivo* voxelwise CBF posterior SDs across all ATTs, which represent the average uncertainties in the CBF estimates, are shown in Fig. 4. The simulation results are shown with both the uniform ATT distribution and weighted by the measured *in vivo* ground truth ATT distribution.

Of the literature protocols, the time-encoded free-lunch approach (Had_free-lunch_) had the lowest average simulation CBF uncertainty across both ATT distributions, including lower than single-PLD and Seq_single-LD_ (4% and 8% lower for the uniform ATT distribution, respectively). Across all the protocols, the novel hybrid approach with *T_1_*-adjusted sub-boluses (Hybrid*_T1_*_-adj_) had the lowest simulation CBF uncertainty (13% and 9% lower than single-PLD and Had_free-lunch_, respectively, for the uniform ATT distribution).

The *in vivo* results match the simulations well, particularly when the *in vivo* ATT distribution is used to weight the simulation results. The upweighting of shorter ATTs found *in vivo* led to several differences, including single-PLD having worse CBF uncertainty than all the multi-timepoint protocols except Had_fixed_ and the performance of Had*_T1_*_-adj_ being improved relative to Had_free-lunch_ and Seq_single-LD_. *In vivo*, Had*_T1_*_-adj_ had the lowest average CBF uncertainty of the literature protocols (16% and 4% lower mean posterior SD than single-PLD and Had_free-lunch_, respectively), while Hybrid*_T1_*_-adj_ maintained the lowest average CBF uncertainty of all the protocols in all cases (24% and 14% lower than single-PLD and Had_free-lunch_, respectively, *in vivo*).

The subjectwise data for all three comparison metrics are shown in Supporting Information Figure S6 and demonstrate similar trends to the voxelwise comparisons, though with fewer significant differences between protocols due to the lower statistical power of these comparisons.

### 4.6 Comparison of protocols: accuracy

**Fig. 5:**
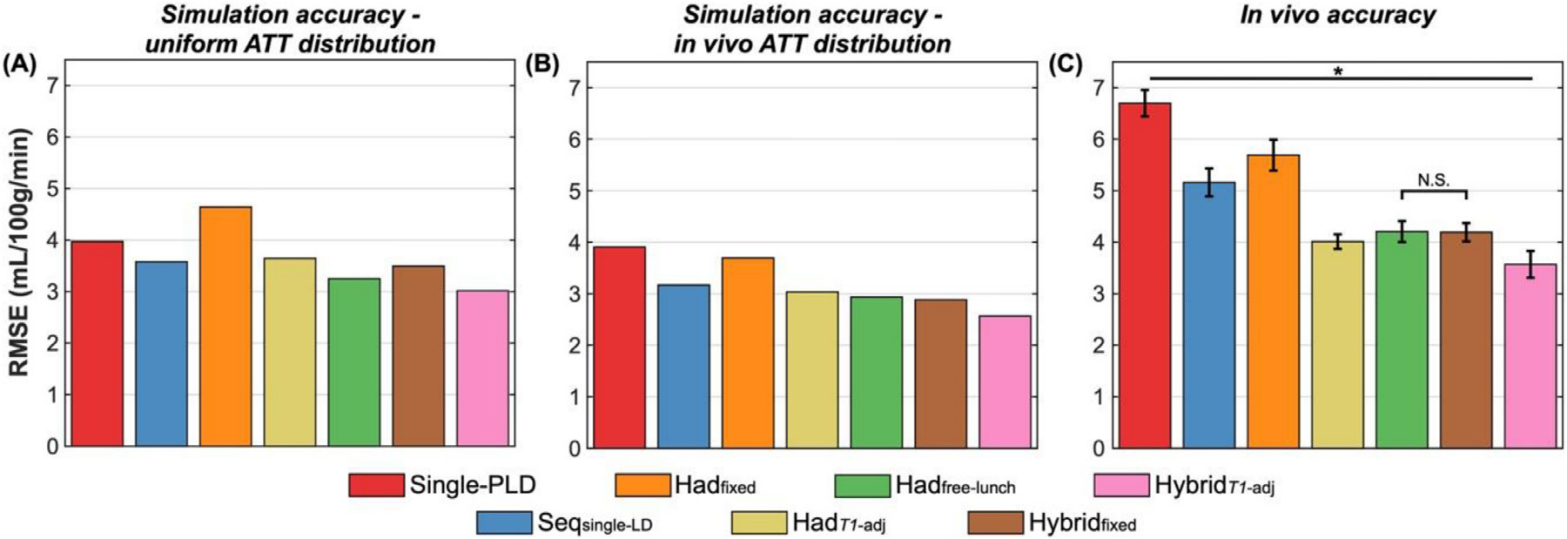
The accuracy of CBF estimates (RMSE relative to ground truth) derived from MC simulations (A, B) and *in vivo* data (C) across all voxels. (A) Shows the simulation results for the uniform ATT distribution, while (B) shows the simulation results weighted by the measured *in vivo* ground truth ATT distribution. *In vivo*, the means and standard errors of the bootstrap distributions are shown (see Methods Section 3.7). All differences were significant except for *Had*_free-lunch_ vs Hybrid_fixed_*in vivo* (two-sided paired Wilcoxon signed-rank test, Bonferroni correction for 21 comparisons, *p* < 0.05).

Fig. 5 shows the simulation and *in vivo* voxelwise RMSEs, which represents a measure of accuracy in the CBF estimates, including both systematic bias and precision, with a lower RMSE meaning a protocol was more accurate. The relative performance of each protocol was similar to that seen with the uncertainty metric used above, with Had_free-lunch_ having the best simulation CBF accuracy of the literature protocols (18% and 9% lower RMSE than single-PLD and Seq_single-LD_ for the uniform ATT distribution, respectively), although Had*_T1_*_-adj_ had the best accuracy *in vivo* (40% and 5% lower RMSE than single-PLD and Had_free-lunch_, respectively). Over all the protocols, Hybrid*_T1_*_-adj_ had the best CBF accuracy in both simulation (24% and 7% lower RMSE than single-PLD and Had_free-lunch_ for the uniform ATT distribution, respectively) and *in vivo* (47% and 15% lower RMSE than single-PLD and Had_free-lunch_, respectively). The accuracy of single-PLD is poorer relative to the multi-timepoint protocols than in the uncertainty comparison due to the bias caused by assuming a fixed ATT, whereas ATT is estimated in the ground truth data and multi-timepoint protocols.

#### 4.6.1 Validation of *in vivo* ground truth estimates

Of course, these *in vivo* results assume that the ground truth CBF estimates, which are generated from all the acquired data fitted simultaneously, are accurate and are not biased towards any particular protocol. The ground truth posterior CBF and ATT SDs were (median ± IQR) 1.14 ± 0.41 mL/100 g/min and 0.017 ± 0.010 s, respectively, suggesting the ground truth estimates were accurate. Supporting Information Figure S7 demonstrates through simulations that, if 3 noise magnitudes are used to estimate the ground truth CBF, as we have done here, there is minimal bias towards any particular protocol in this comparison.

### 4.7 Comparison of protocols: repeatability

**Fig. 6:**
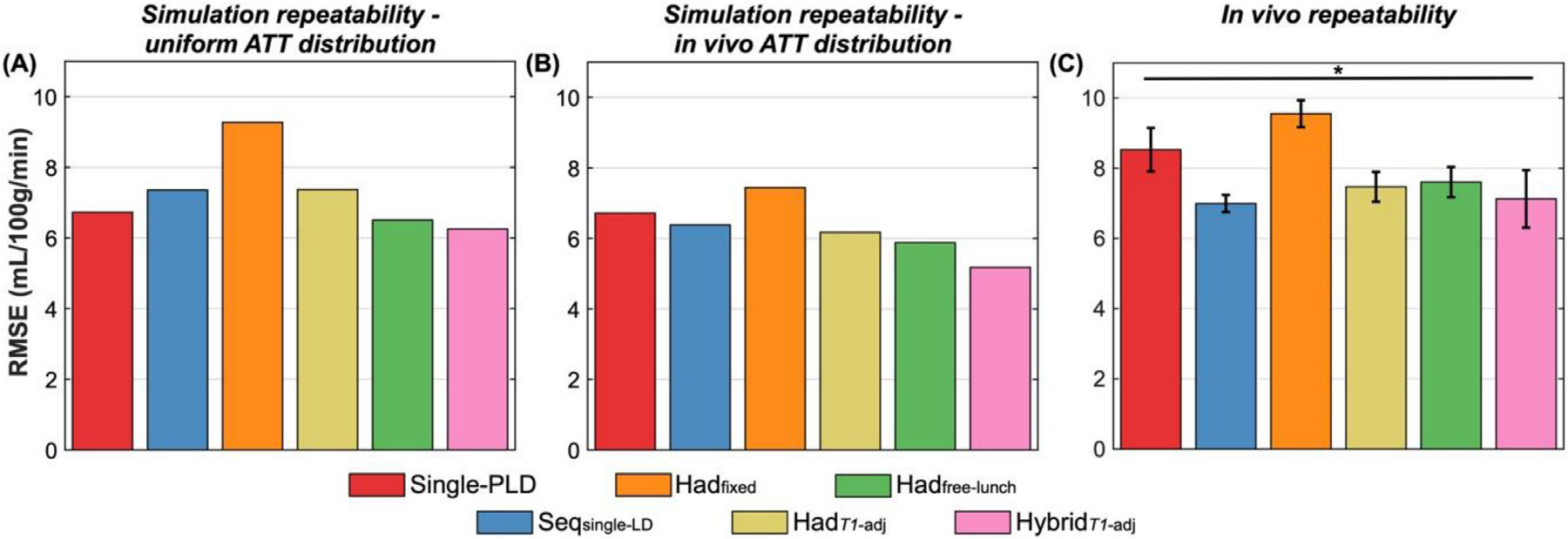
The repeatability of CBF estimates (test-retest RMSEs) derived from MC simulations (A, B) and *in vivo* data (C) across all voxels. (A) Shows the simulation results for the uniform ATT distribution, while (B) shows the simulation results weighted by the measured *in vivo* ground truth ATT distribution. *In vivo*, the means and standard errors of the bootstrap distributions are shown (see Methods Section 3.7). All differences were significant *in vivo* (two-sided paired Wilcoxon signed-rank test, Bonferroni correction for 15 comparisons, *p* < 0.05).

The mean MC simulation and *in vivo* test-retest voxelwise CBF RMSEs are shown in Fig. 6. A lower test-retest RMSE means a protocol was more repeatable. Note, a further 2.4% of the *in vivo* GM voxels were excluded from this comparison because one or more of the 2.5 min scans had CBF or ATT posterior SDs >500 mL/100 g/min or >50 s, suggesting very poor fits.

The simulation results again reflect those of the uncertainty metric, with Had_free-lunch_ having the best repeatability of the literature protocols (3% lower test-retest RMSE than single-PLD for the uniform ATT distribution) while Hybrid*_T1_*_-adj_ had the best repeatability overall (7% and 4% lower test-retest RMSE than single-PLD and Had_free-lunch_, respectively, for the uniform ATT distribution). This again demonstrates that more robust CBF estimates can be obtained with certain multi-timepoint protocols than a single-PLD protocol, in this case using a metric which is not reliant on uncertainty estimates from the fitting algorithm nor any estimated ground truth.

As before, there were differences due to the shorter average ATTs seen *in vivo* than were simulated for the uniform ATT distribution, causing the *in vivo* single-PLD CBF repeatability to be worse relative to the multi-timepoint protocols. Another result also only seen *in vivo* was that Seq_single-LD_ had the best repeatability of all the protocols (RMSE = 7.00±0.24 mL/100 g/min), better than Hybrid*_T1_*_-adj_ (RMSE = 7.13±0.82 mL/100 g/min). However, the subjectwise analysis, shown in Supporting Information Figure S6(C), demonstrates that there was one subject with much higher CBF test-retest RMSE for Hybrid*_T1_*_-adj_ than the other subjects. There was an average GM CBF increase of 10 mL/100 g/min between the two halves of the Hybrid*_T1_*_-adj_ scan for this subject, possibly due to a change in subject alertness (Clement et al., 2018). After removing this subject from the comparison, Hybrid*_T1_*_-adj_ had the best CBF repeatability across all protocols (test-retest RMSE = 6.33±0.41 ml/100 g/min: 28% and 15% lower than single-PLD and Had_free-lunch_, respectively) while the Seq_single-LD_ repeatability was relatively unaffected (7.01±0.27 mL/100 g/min) (see Supporting Information Figure S8).

### 4.8 Comparison of protocols: arterial transit time

Although the protocols were not optimized for ATT accuracy, the results of the ATT comparisons are briefly described here. The *in vivo* voxelwise measures of ATT uncertainty, accuracy, and repeatability are shown in Supporting Information Figure S9 and demonstrate that the time-encoded and hybrid protocols all have more confident, accurate, and repeatable ATT estimates than Seq_single-LD_. Had*_T1_*_-adj_ had the lowest uncertainty and best repeatability, while Had*_T1_*_-adj_ and Hybrid*_T1_*_-adj_ both had the highest accuracy.

### 4.9 Had_variable_ and Hybrid_variable_protocols

The optimal Had_variable_ and Hybrid_variable_ protocol timings are given in Table 2 and the MC simulation uncertainties are shown in Supporting Information Figure S10. The Had_variable_ timings and uncertainties are similar to those of the Had_free-lunch_ protocol, though the average uncertainty is slightly lower for Had_variable_. Similarly, the optimized Hybrid_variable_ protocol only provided a small reduction in uncertainty relative to Hybrid*_T1_*_-adj_. These results suggest that the constraints of the Had_free-lunch_ (with *T_1_*-adjusted LDs) and Hybrid*_T1_*_-adj_ protocols are near optimal within their respective class of protocols, making them attractive protocol designs due to the reduced optimization complexity resulting from their timing constraints. For these reasons, Had_variable_ and Hybrid_variable_ were not included during the *in vivo* comparison.

## 5 Discussion

### 5.1 Summary of findings

In this study, we optimized a wide range of PCASL protocol designs for CBF accuracy, using a previously developed Cramér-Rao Lower Bound based optimization algorithm, and compared their CBF estimates using Monte Carlo simulations and *in vivo* experiments, which were in good agreement. The CBF estimates were compared with: (1) the standard deviation of the marginal posterior probability distributions from the fitting algorithm as a measure of uncertainty; (2) the RMSEs of the estimates relative to the ground truth estimates as a measure of accuracy, which includes both random variability and systematic biases; and (3) the RMSEs of the test-retest estimates as a measure of repeatability.

We demonstrated that the novel hybrid protocol with *T_1_*-adjusted sub-bolus durations (Hybrid*_T1_*_-adj_) had the most confident, most accurate and most repeatable CBF estimates of all the tested protocols, including the single-PLD protocol and the literature sequential and time-encoded multi-timepoint protocols. This highlights the benefit of generating multi-timepoint ASL data from both time-encoded sub-boluses and sequential PLDs. This hybrid method benefitted from the longer LDs possible with a smaller encoding matrix, but still achieved a time-decoding noise reduction factor of 2 and maintained a sufficiently well sampled range of unique PLDs due to the use of multiple sequential PLDs.

These results also highlight that, even though the multi-timepoint protocols have lower SNR at each timepoint compared to the single-PLD protocol, some can achieve more accurate CBF estimates on average across a range of ATTs. This is because the noise in multi-timepoint data is essentially averaged across the data during the fitting process, resulting in similar noise averaging to the single-PLD protocol, but with data that more effectively samples the signal curve across the range of ATTs. Multi-timepoint protocols also have the added benefit of generating ATT maps, which is an interesting physiological parameter in its own right.

Of the protocol designs from the literature, the free-lunch time-encoded protocol (Had_free-lunch_) with *T_1_*-adjusted sub-bolus durations was found to have CBF estimates that were more confident, accurate, and repeatable than the other literature designs, including the single-PLD protocol, for the uniform ATT distribution used in the simulations. Due to the shorter average ATTs witnessed *in vivo*, however, the Had*_T1_*_-adj_ protocol outperformed Had_free-lunch_. It was seen in simulation that the sequential multi-PLD protocol (Seq_single-LD_) produced similarly confident, accurate, and repeatable CBF estimates on average to Had*_T1_*_-adj_. This suggests that the averaging benefit from time-decoding for Had*_T1_*_-adj_ is similar to the benefit of longer LDs and more flexible PLDs for Seq_single-LD_. It is also apparent that the use of fixed-LDs in time-encoded PCASL is a sub-optimal design for CBF estimation.

### 5.2 Comparison of protocols: using long label durations

The longest LD used in this work was 1.8 s, which is currently recommended for clinical use with a single-PLD (Alsop et al., 2015). It has been suggested that it is more SNR efficient to use long LDs of 3–4 s (resulting in fewer averages) for single-PLD PCASL (Zun et al., 2014) with additional benefits of reduced temporal signal variation and reduced sensitivity to delayed ATTs (Dai et al., 2012; Lebel et al., 2015). To investigate the extent to which the CBF accuracy of the protocols in this work may benefit from longer LDs, we repeated the protocol optimizations with a maximum LD of 5 s and conducted further MC simulations as before. The LD of the single-PLD protocols was optimized similar to (Zun et al., 2014) but for a 5 min scan, a PLD of 2 s, and an ATT range 0.5 - 2 s.

**Fig. 7:**
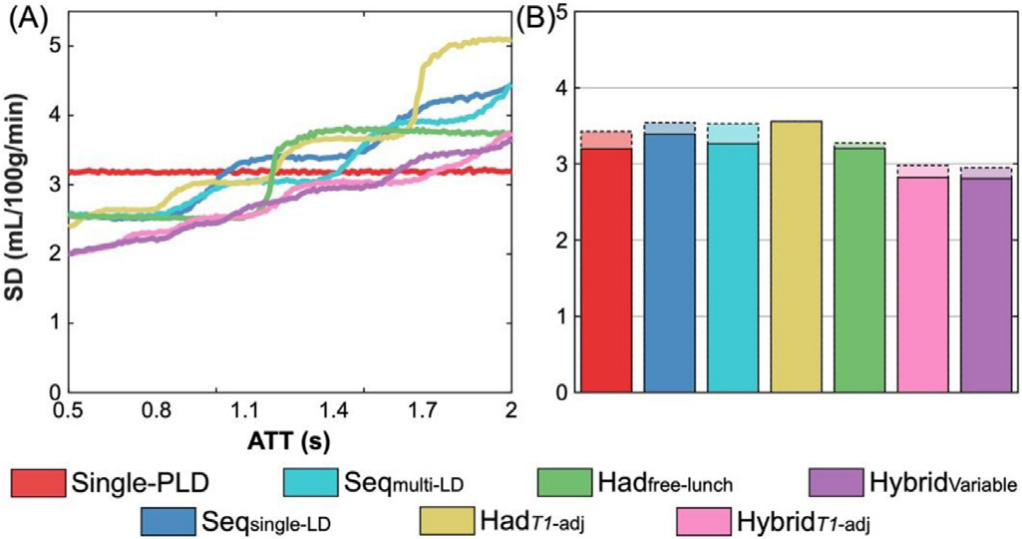
The MC simulation CBF posterior SDs for a selection of the protocols optimised with a maximum LD of 5 s. (A) the median posterior SDs for each protocol across ATTs, (B) the mean posterior SDs for each protocol across the ATT range. In (B), the mean posterior SDs for the short LD cases (maximum LD 1.8 s) are shown as faded bar graphs to demonstrate the achieved reductions in uncertainties.

The optimized protocol timings are given in Supporting Information Table S2 and the MC simulation fitting posterior distribution SDs are shown in Fig. 7. All the protocols used much longer LDs than when the maximum LD was 1.8 s, except Had*_T1_*_-adj_ which had the same timings as before. The increase in the protocols' LDs led to an average reduction in the CBF posterior SDs of 0.17 ± 0.07 mL/100 g/min (5.1% ± 1.9%). As before, the Hybrid*_T1_*_-adj_ and Hybrid_variable_ protocols had similar posterior SDs, which were the lowest of all the protocols, including single-PLD. It is possible that the *in vivo* benefits of using longer LDs extend beyond the theoretical benefits found here (Dai et al., 2012; Lebel et al., 2015) and should be investigated further.

### 5.3 Protocol optimization

#### 5.3.1 Optimizing for CBF accuracy

This study was restricted to protocols optimized solely for CBF accuracy. It is also possible to simultaneously optimize for CBF and ATT accuracy (Owen et al., 2016; Sanches et al., 2010; Santos et al., 2010; Woods et al., 2019; Xie et al., 2008) but we chose to focus on CBF estimation for two reasons: 1) CBF is often the main parameter of interest, with knowledge of ATT predominantly being used to correct ATT related biases in the CBF estimates, and 2) optimizing for only one parameter makes interpretation of the final protocols and their relative parameter estimation accuracy simpler. However, there is nothing to prevent the optimization framework being used to also, or solely, optimize for ATT accuracy (Woods et al., 2019).

#### 5.3.2 Single-PLD

The single-PLD protocol used in this study was not optimized using the CRLB framework, as used for the multi-timepoint protocols. The single-PLD protocol is used to estimate one model parameter, CBF, with the ATT fixed at an assumed value (1.3 s in this work). In this case, the CBF uncertainty is proportional to the noise SD, scaled to units of mL/100 g/min by experimentally imposed or fixed literature parameters (see Supporting information text 2 for the single-PLD CBF uncertainty formula). This is in contrast to the multi-timepoint protocols where both CBF and ATT are estimated, meaning the CBF uncertainty also depends on the covariance between CBF and ATT and the estimated ATT, leading to variation across ATTs. Use of this optimization framework with the single-PLD protocol would minimize the protocol's CBF variance across the ATT distribution, but the resultant shorter PLD would result in potentially large CBF underestimation in regions where ATT > PLD (Guo et al., 2018). However, future work could investigate the tradeoff between the accuracy and precision of the single-PLD protocol with a shorter PLD in comparison to the best performing multi-timepoint protocols presented in this study.

#### 5.3.3 Sequential protocols

The optimized Seq_multi-LD_ protocol included only one LD shorter than 1.8 s, suggesting that it is not optimal to use short LDs with short PLDs for CBF estimation for the investigated ATT range, a technique previously used in the literature (Johnston et al., 2015; Zhao et al., 2015). It also does not appear optimal to perform multi-timepoint acquisitions by only varying the LD (Borogovac et al., 2010).

#### 5.3.4 Time-encoded protocols

Although a 4 × 3 encoding for the Had_fixed_ protocol was optimal for CBF estimation, we did not include it in the *in vivo* comparison due to the more common use of 8 × 7, or larger, encodings in the literature (Dai et al., 2013; Günther, 2007; Guo et al., 2018; Teeuwisse et al., 2014; von Samson-Himmelstjerna et al., 2016; Wells et al., 2010). Supporting Information Figure S2 shows the simulation uncertainty and accuracy comparison results when including the optimized 4 × 3 Had_fixed_ protocol (Had_fixed4×3_). Had_fixed4×3_ had lower CBF uncertainty and higher accuracy than Had_fixed8×7_ across most of the ATT range. For the uniform ATT distribution, for which the protocols were optimized, Had_fixed4×3_ had the least confident and least accurate CBF estimates of all the protocols on average, except Had_fixed8×7_. For the *in vivo* ATT distribution, due to the shorter ATTs encountered, Had_fixed4×3_ had more confident CBF estimates than the other literature protocols, but not the Hybrid protocols, and had more accurate CBF estimates than single-PLD and Seq_single-LD_. These results suggest that if a Had_fixed_ protocol is to be used, a 4 × 3 encoding will generate more accurate CBF estimates, which is in line with previous findings (Guo et al., 2017). However, for the intended ATT distribution, a *T_1_*-adjusted Hadamard design is still a superior alternative, while the Hybrid*_T1_*_-adj_ protocol remained superior at almost all ATTs, making it extremely robust to ATT variations within the optimized range.

The standard time-encoded protocols were relatively simple and fast to optimize, due to the reduced dimensionality of the timing parameter space enforced by the design constraints. This contrasts with the sequential and hybrid protocols which must be iteratively optimized, and therefore take more time; the Seq_mutli-LD_, Had_variable_, and Hybrid_variable_ protocols also required many random initializations to avoid local minima. Since the optimization only needs to be performed once, this is not a major drawback. However, the standard time-encoded protocols might make better candidates for real-time protocol optimization since they can be quickly adjusted during a scan to better match patient specific ATT information generated from preceding TRs (Xie et al., 2010).

Hadamard-encoding schemes were used for the time-encoded protocols because these provide the most efficient encodings. However, they can only be of size (rows × columns) 2k × (2k-1), for *k* = 1,2,4,6,8,10,…. Less efficient encodings may provide more flexibility in the protocol timings and could be explored with the same optimization framework used in this work.

Time-encoded protocols which rely on a decoding step are potentially more susceptible to motion and physiological artefacts, since more images are used to decode each difference image than standard ASL protocols. Although several methods have been proposed to improve the robustness of these methods to data corruption, including Walsh-ordered Hadamard encodings (von Samson-Himmelstjerna et al., 2016) and subtraction free CBF estimation, which can exclude corrupted volumes (von Samson-Himmelstjerna et al., 2015), future studies could investigate whether it is beneficial or not to use the optimized time-encoded protocols developed in this work compared to optimized sequential multi-timepoint and single-PLD protocols in the context of missing data. The optimized Hybrid*_T1_*_-adj_ protocol only uses a 4 × 3 encoding matrix and so should be more robust to motion than protocols which use larger encodings.

### 5.4 Choice of ATT prior distribution

A uniform ATT prior distribution representative of gray matter in young healthy volunteers of 0.5–2 s was chosen based on the ATT range seen in (Woods et al., 2019), which used a similar labeling plane placement. However, the *in vivo* ATTs in this study were generally shorter, with 95% of the ground truth ATTs ≤1.51 s. This may be due in part to the use of a visual stimulus to maintain subject alertness, which can lead to a reduction in ATTs in the visual cortex (Qiu et al., 2010), a region which typically has longer ATTs than other GM brain regions (Dai et al., 2017). Flow crushing gradients were also not used here, which have been shown to increase the measured ATTs across the brain (Dai et al., 2017), though the spoiler gradients sandwiching the GRASE refocusing pulses will have caused some flow crushing (Günther et al., 2005). It should also be noted that 5.2% of the voxels had ATTs <0.5 s, which was outside the optimized ATT range. If these voxels are excluded from the analysis, however, the results are similar and the conclusions remain unchanged (results not shown). Since resting ASL scans do not typically use a visual stimulus and vascular crushing is not currently recommended for clinical scans (Alsop et al., 2015), it is likely an ATT prior range of 0.5–1.8 s is sufficient for protocol optimization for young healthy volunteers. However, a range of 0.5–2 s may be more appropriate if vascular crushing is used or for older populations (Dai et al., 2017).

In cases where the ATT is likely to be greatly delayed relative to the range chosen in this work, the protocols should ideally be optimized for a longer range of ATTs, as demonstrated in (Woods et al., 2019) for sequential multi-PLD protocols. Although optimizing for longer ATTs will improve the CBF estimation accuracy in these cases, this comes at the cost of lower accuracy at short ATTs. The CBF accuracy at long ATTs is also inherently limited compared to that of short ATTs due to increased *T_1_* decay of the label. Future studies could compare the performance of the best protocols from this work in the case of delayed ATTs.

### 5.5 Use of *in vivo* ground truth estimates

For the *in vivo* accuracy comparison (Section 4.6), in the absence of an independent gold standard CBF measurement we used *in vivo* ground truth CBF estimates derived from fitting the combined ASL data. This assumes that these ground truth estimates are accurate and not greatly affected by bias. These assumptions were confirmed by the small ground truth posterior probability SDs and the relatively equal bias found in simulations across protocols.

However, deviations from the model parameters derived from literature values could lead to biases in the CBF estimates which differentially affect each protocol and the ground truth estimates. Parameters which scale the magnitude of the ASL signal, such as the blood *T_1_* and labeling efficiency, will similarly affect each protocol. The tissue *T_1_* (*T_1t_*) and brain/blood water partition coefficient (λ), though, vary the shape of the kinetic curve. Variations in λ will have a negligible effect under normal physiological conditions (Buxton et al., 1998), but errors in *T_1t_* could lead to different CBF biases across protocols. Supporting Information Figure S11 shows, using simulations, how variations in the true *T_1t_* affect the accuracy of the protocols relative to the estimated ground truth values. We found that when the true *T_1t_* varies, but the fitting *T*_*1t*_ is fixed, the relative accuracy of the protocols remains very similar, except for the single-PLD protocol. This suggests that reasonable variations in *T_1t_* would not greatly affect the results of the *in vivo* accuracy comparison

Additionally, the good agreement between the simulation accuracy comparison, where the ground truth is known, and *in vivo* comparison further suggests the validity of the ground truth estimates in this study.

### 5.6 Variable CBF uncertainty across ATTs

There was a gradual increase in the *in vivo* CBF uncertainty (posterior distribution SDs) at shorter ATTs compared to the simulations, which assumed equal noise across all ATTs. One explanation is that brain regions with shorter ATTs are generally located closer to the middle of the brain and so further from the head-coil receive elements than regions with longer ATTs. This could result in an SNR level that was negatively corelated with ATT. Another explanation is that shorter ATTs are in regions closer to larger upstream arteries and so experience greater signal variability due to cardiac pulsation.

To test the hypothesis that the CBF posterior SDs vary with ATT, the voxelwise temporal noise, σ, was calculated from the calibrated *in vivo* single-PLD control images by taking the SD across repeats. A linear model, σ(ATT)=a·ATT+b, was fit to these data from all subjects using the ground-truth ATT estimates and the "fit" function in MATLAB using bisquare weights, which is robust to outliers. The fitted parameters were $a=-4.28\;\times 10^{-4}\;$s^−1^and $b=20.29\;\times 10^{-4}$with the model explaining 58% of the variance $\left(R^{2}=0.58\right)$, indicating there is indeed increased noise in the control images at locations with shorter ATTs.

**Fig. 8:**
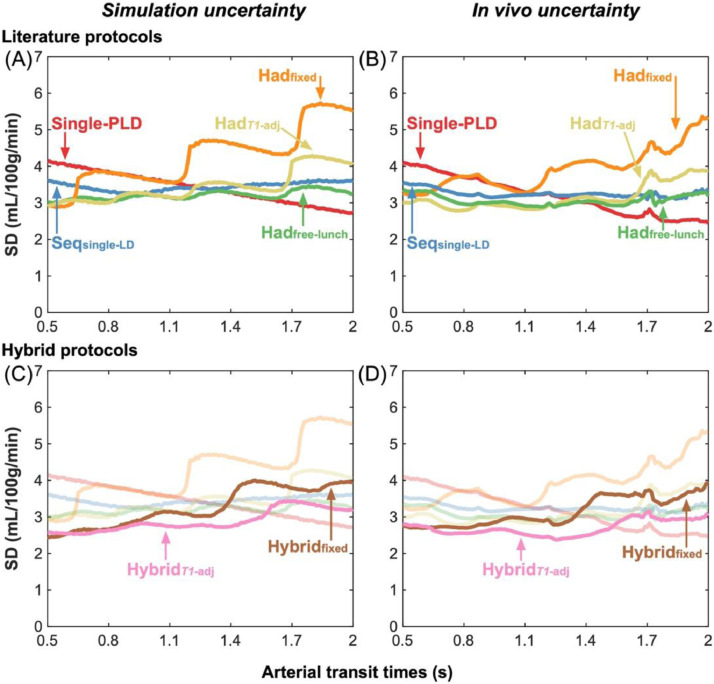
The variable noise MC simulation (posterior SDs) (A, C) and *in vivo* (posterior SDs) (B, D) median CBF uncertainty measures for the literature protocols (top) and the proposed hybrid protocols (bottom). These MC simulations use the estimated variable noise levels across ATTs calculated from the *in vivo* single-PLD data and scaled to the noise SD used in the original MC simulations. The variable noise MC simulation uncertainty trends across ATTs better match the trends seen *in vivo*.

This noise model was used in additional MC simulations, similar to those described in the Methods Section 3.2, after being rescaled so that *σ*(1.25 s) was equal to the noise SD used in the original simulations. Fig. 8 shows a comparison between the variable noise simulations and *in vivo* data, demonstrating a much-improved qualitative match than the fixed noise simulations. This suggests the relationship between ATT and temporal signal variation largely explains the differences seen in the trends *in vivo*, though we cannot deduce the relative contributions to this variability from pulsatility or proximity to the receive coil using our data. This ATT dependent noise model may not be useful for general protocol optimization because it will likely vary across subjects, subject placement, and head coil design.

### 5.7 CBF and ATT variation across subjects

Large differences in the CBF and ATT maps were seen across subjects (Supporting Information Figure S3 and Supporting Information Figure S4). These differences may be due to previously seen global variations across age and sex, such as decreasing CBF and increasing ATT with age (Chen et al., 2011; Dai et al., 2017; Parkes et al., 2004) and higher CBF and lower ATT in women (Henriksen et al., 2013; MacIntosh et al., 2010; Vernooij et al., 2008).

### 5.8 Macrovascular signal

We did not use vascular crushing in this study due to its incompatibility with the 3D GRASE readout and the increased signal loss with a separate vascular crushing module. Although there is some inherent vascular crushing in the GRASE readout (Günther et al., 2005), there will be residual macrovascular signal in these data, particularly at short PLDs, which may lead to errors in the CBF estimates.

In this work, we used a one-compartment model to fit the data because the introduction of more free model parameters with a two-compartment model leads to increased CBF and ATT uncertainties. This would not be a problem if there were a uniform increase in the uncertainties, however, when we used a two-compartment model, the uncertainties increased by varying amounts across ATTs in a different way for each protocol. This is due to the different temporal sampling of each protocol. The fast dynamics of the macrovascular compartment are best fit to high-temporally sampled data (van der Plas et al., 2019), but the density of the timepoints varies greatly within and across the protocols. This can lead to high uncertainties on the macrovascular compartment which greatly increase the uncertainties of the CBF and ATT estimates.

In order to exclude voxels with macrovascular signal from biasing the comparison, we used the posterior distribution SDs output by the fitting algorithm to identify voxels which were not well fit by the one-compartment model. As Supporting Information Figure S5 shows, the excluded voxels were mostly located in regions with large arteries, suggesting that our goal was successful. Furthermore, the good agreement in the relative performances of the protocols between simulation and *in vivo* suggests that any remaining residual macrovascular effects have not dominated the *in vivo* comparison.

## 6 Conclusions

In this work, we demonstrated that optimized multi-timepoint protocols can generate more confident, accurate, and repeatable CBF estimates across a given ATT range than a single-PLD protocol, while also generating ATT maps. We found that the time-encoded free-lunch protocol with *T_1_*-adjusted LDs can lead to improved CBF estimates over a fixed-LD time-encoded protocol and is a good approximation to the optimal time-encoded design. Finally, we demonstrated that a novel hybrid time-encoded with sequential PLD protocol design utilizing *T_1_*-adjusted label durations out-performed a wide range existing literature protocol designs for estimating CBF, both in simulation and *in vivo*.

## CRediT authorship contribution statement

**Joseph G. Woods:** Conceptualization, Methodology, Software, Formal analysis, Investigation, Data curation, Writing - original draft. **Michael A. Chappell:** Conceptualization, Writing - review & editing, Supervision. **Thomas W. Okell:** Conceptualization, Resources, Writing - review & editing, Supervision.

## Declarations of Competing Interest

None

## Appendix Supplementary materials

Image, application 1
