# Supplementary material for: Designing and comparing optimized pseudo-continuous Arterial Spin Labeling protocols for measurement of cerebral blood flow
Source: Neuroimage. 2020 Dec;223:117246. doi: 10.1016/j.neuroimage.2020.117246 (PMC7762814; doi:10.1016/j.neuroimage.2020.117246)
Supplement: Supplementary file 1 [file mmc1.pdf]

Supporting information text 1 - protocol optimization

Optimization updates

Details of the optimization implementations are as follows. To optimize the $Seq_{single\text{-}LD}$ protocol, the PLDs were optimized in the same way as the original implementation, but the optimal single experiment LD was found when optimizing the final (N_{th}) PLD. For the $Seq_{multi\text{-}LD}$ protocol, the optimal i_{th} LD and PLD pair were found at each iteration. In both cases, the PLDs were restricted to a monotonically increasing order ($PLD_{i-1} \leq PLD_i \leq PLD_{i+1}$) to reduce the parameter space.

Due to the design constraints of the Had_{fixed} and $Had_{T1\text{-}adj}$ protocols, only the first LD and final PLD, for any encoding size, $(M + 1) \times M$, must be searched over, making it possible to carry out a global grid search for all possible timing combinations for each M, rather than use the iterative exchange method used with the sequential protocols. The $Had_{free\text{-}lunch}$ protocol differs only in that the first encoded LD is fixed to the single-PLD protocol LD, with the remaining LDs being optimized identically to the Had_{fixed} and $Had_{T1\text{-}adj}$ protocols.

The $Hybrid_{fixed}$ and $Hybrid_{T1\text{-}adj}$ protocols were optimized by iterating through each of the sequential N PLDs and optimizing the i_{th} PLD and LDs of the encoding matrix simultaneously.

$Had_{variable}$ was optimized by iterating through the encoded LDs and simultaneously optimizing the i_{th} LD and the final PLD. $Hybrid_{variable}$ was optimized by iterating through each of the sequential N PLDs and encoding matrices and then iterating through each of the M LDs, optimizing the j_{th} LD of the i_{th} encoding matrix with the i_{th} PLD together. Initial testing of these variable-LD protocols suggested the best protocols had LDs of decreasing duration during the PCASL preparation, so the LD was restricted to $LD_{j-1} \leq LD_j \leq LD_{j+1}$.

Protocol initializations

$Seq_{single\text{-}LD}$: initialized with a single LD of 1.8 s and N PLDs spaced evenly between 0.075 - 2.3 s. $Seq_{multi\text{-}LD}$: N LD and PLD pairs randomly initialized between 0.8 - 1.8 s and 0.075 - 2.3 s,

respectively. For each N, the sequential protocol optimizations iterated through each timepoint in a randomly permuted manner and were run with 20 different initializations for robustness.

Had$_{free\text{-}lunch}$: the first LD was fixed at 1.8 s (matching the single-PLD protocol) with the remaining LDs being either fixed-duration or T_1-adjusted. The final PLD was also optimized, therefore, it was not guaranteed that the PLD of the first encoded LD would match that of the single-PLD protocol, which it does not (see Table 2). The Had$_{fixed}$ and Had$_{T1\text{-}adj}$ protocols did not require initialization because the entire timing parameter space could be evaluated.

Hybrid$_{fixed}$ and Hybrid$_{T1\text{-}adj}$: all N final PLDs initialized at 0.075 s - the LDs did not require initialization because they are all globally optimized at each step, similar to the time-encoded protocols.

Had$_{variable}$ and Hybrid$_{variable}$: the LDs were randomly initialized between 0.1 - 1.8 s and sorted into a descending order; the N PLDs were initialized to 0.075 s. In the case of Hybrid$_{variable}$, the N PLDs were iterated through in the same order. For both Had$_{variable}$ and Hybrid$_{variable}$, the M LDs were iterated through in a randomly permuted order. The optimizations were each run with 50 different initializations for robustness.

Supporting information text 2 - single-PLD CBF Cramér-Rao lower bound

If the PLD is longer than the assumed ATT, as is the case in this work, the CBF uncertainty (Cramér-Rao lower bound standard deviation) for the single-PLD protocols is:

$$\sigma_f = \sqrt{F^{-1}} = \sqrt{\left(\frac{A}{\sigma^2}\sum_{i=1}^{N}\left(\frac{\partial \Delta M(t_i;f;\rho)}{\partial f}\right)^2\right)^{-1}} = \frac{\sigma}{\sqrt{A}} \cdot \sqrt{\frac{e^{\frac{\Delta t}{T_{1b}}} e^{\frac{t-\tau-\Delta t}{T_1'}}}{2M_{0B}T_1'\alpha\left(1-e^{-\frac{\tau}{T_1'}}\right)}} \quad .$$

When Δt, the ATT, is fixed, σ_f does not vary with the underlying true ATT.

In the above equation, f is the CBF, σ_f is the CBF uncertainty, F is the Fisher information matrix, A is the number of averages, σ is the noise standard deviation, ΔM is the ASL difference signal which depends on t_i (the i_{th} acquired timepoint), f, and ρ (the remaining model parameters), and $\frac{\partial \Delta M}{\partial f}$ is the CBF sensitivity function, the formula for which is derived in Woods et al., 2019. For the parameters in the CBF sensitivity function, Δt is the ATT (assumed to be 1.3 s for this protocol), τ is the labeling duration, M_{0B} is the equilibrium blood magnetization, α is the labeling efficiency, T_{1b} is the blood T_1, and $\frac{1}{T_1'} = \frac{1}{T_{1t}} + \frac{f}{\lambda}$, where T_{1t} is the tissue T_1 and λ is the brain-blood water partition coefficient.

Supporting information figures and tables

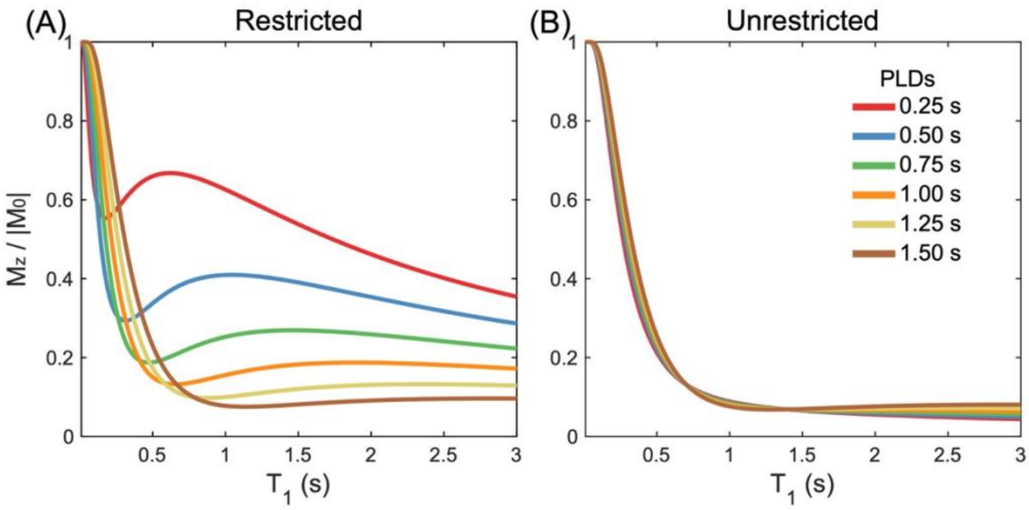

Supporting Information Figure S1: The theoretical residual static tissue longitudinal magnetisation at the time of the readout excitation. The BGS uses a presaturation module and two inversion pulses to. null T_1 = 700 ms and 1400 ms. (A) The residual magnetisation when the inversion pulses are restricted to play out after the LD; (B) the residual magnetisation when the inversion pulses are played out at the optimal times, including during the LD. For both (A) and (B), the residual longitudinal magnetisation is shown for 6 different PLDs (0.25 - 1.5 s) and a LD of 1.4 s. Instantaneous RF pulses, perfect spoiling, and perfect inversion are assumed. The null time has been set to 100 ms before the excitation, to ensure positive signal in all cases.

Protocol	***Label durations (ms)***	***Post-label delays (ms)***	N_T	N_{Ave}	N_{Acq}	***Scan duration (min)***
***Seq**multi-LD*	1800, 1500, 1800, 1800, 1800, 1800, 1800, 1800, 1800, 1800, 1800, 1800	200, 825, 1350, 1475, 1800, 1850, 2100, 2225, 2300, 2300, 2300, 2300	12	3	72	5:00
Had**fixed* ***4×3	1125, 1125, 1125	75	3	18	72	4:54

Supporting Information Table S1: The optimized protocol timings for $Seq_{multi\text{-}LD}$ and Had_{fixed} with a 4×3 Hadamard matrix, which were not included in the in vivo comparison. N_T is the number of effective PLDs, N_{Ave} is the number of averages, and N_{acq} is the number of acquired volumes for each scan.

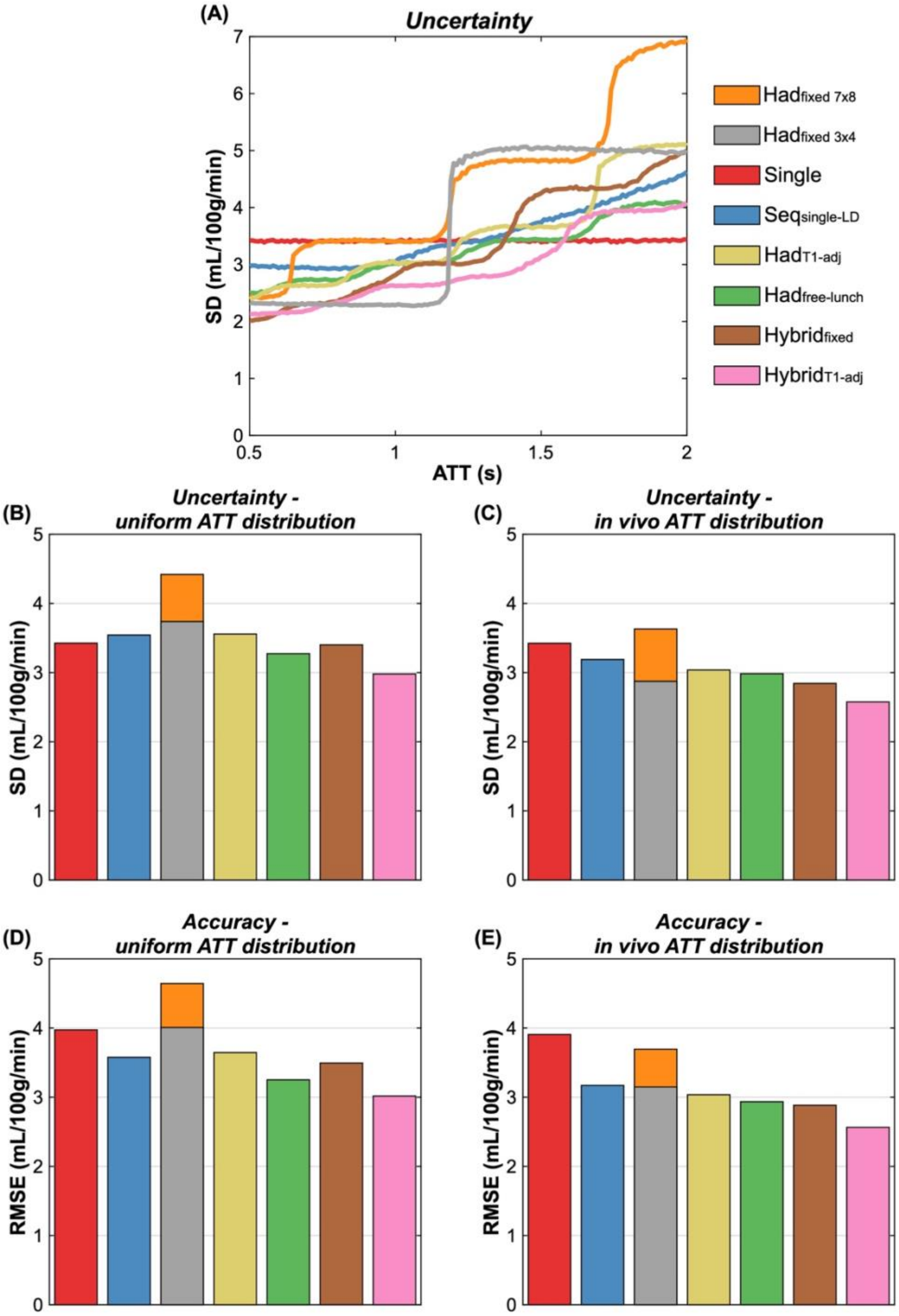

Supporting Information Figure S2: The MC simulation CBF uncertainty (mean posterior SDs) and accuracy (RMSE relative to ground truth) for the optimised Had$_{fixed}$ protocol when using a 4×3 or 8×7 Hadamard matrix. (A) the median uncertainty across ATTs, (B) the mean uncertainty with a uniform ATT distribution, (C) the mean uncertainty with the in vivo ATT distribution, (D) the mean accuracy with the uniform ATT distribution, (E) the mean accuracy with the in vivo ATT distribution.

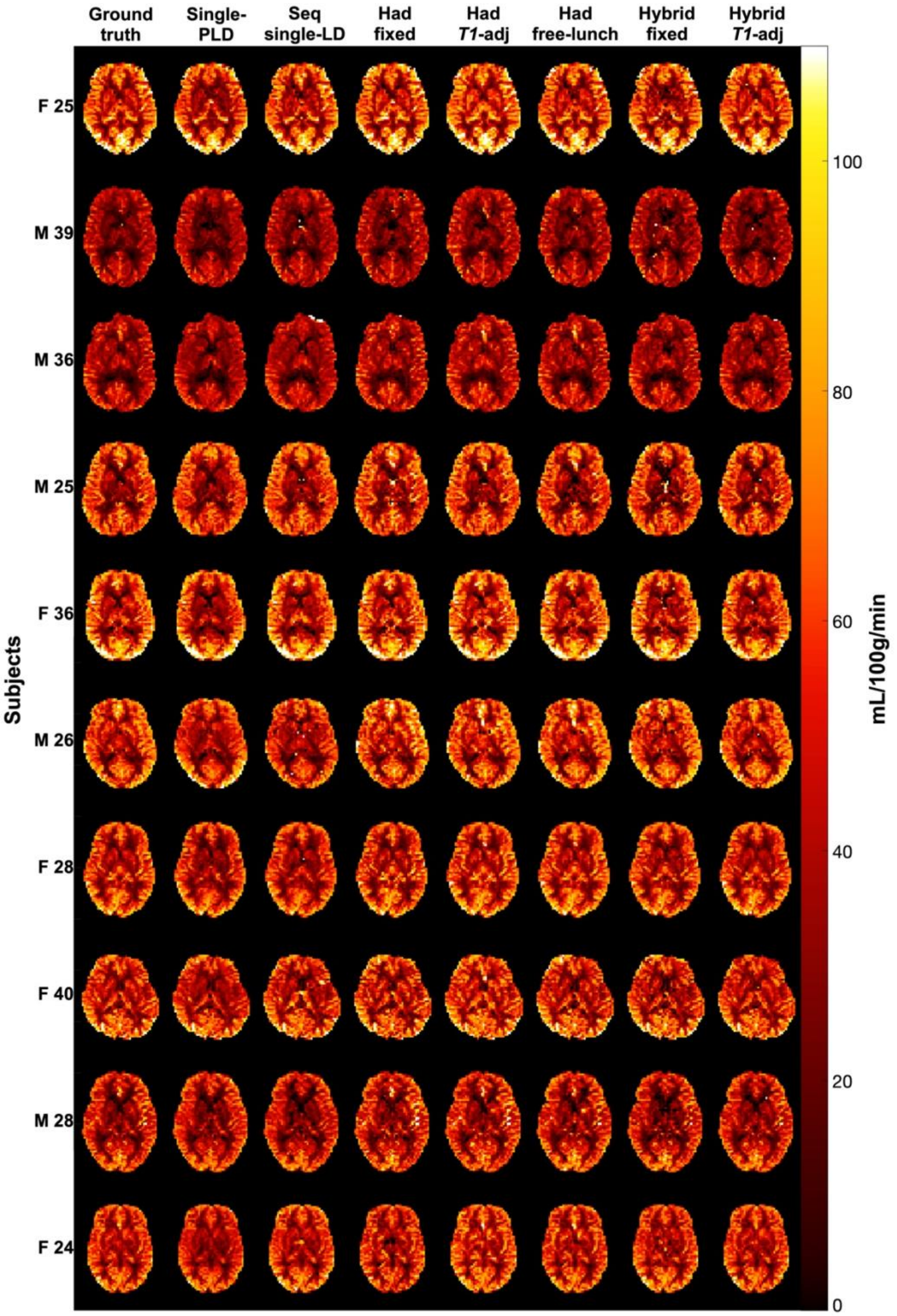

Supporting Information Figure S3: A single slice of the CBF maps for each subject and each protocol. The subjects' sex and age are given, where "F 25" means "Female, 25 years old."

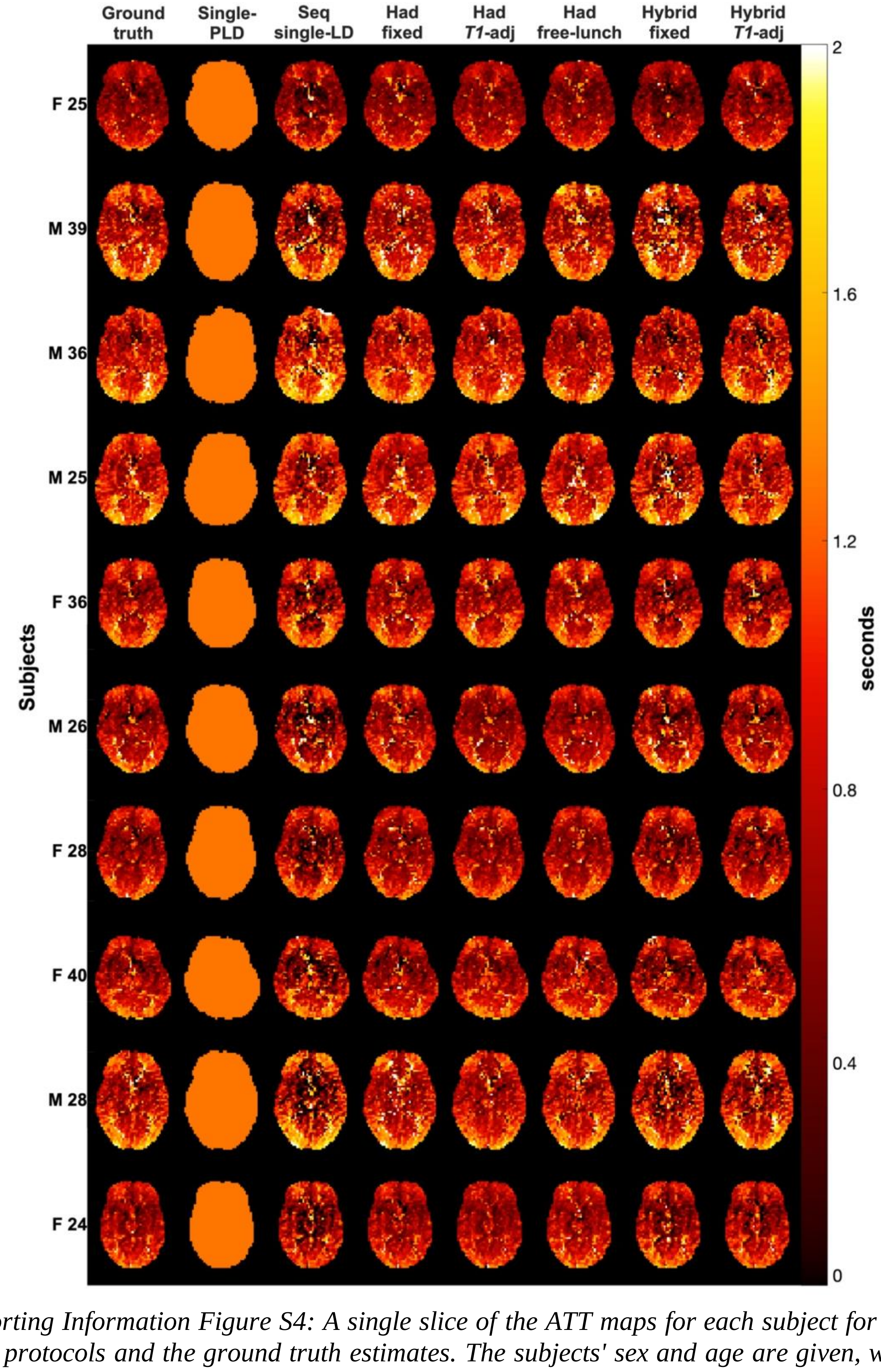

Supporting Information Figure S4: A single slice of the ATT maps for each subject for each of the protocols and the ground truth estimates. The subjects' sex and age are given, where "F 25" means "Female, 25 years old."

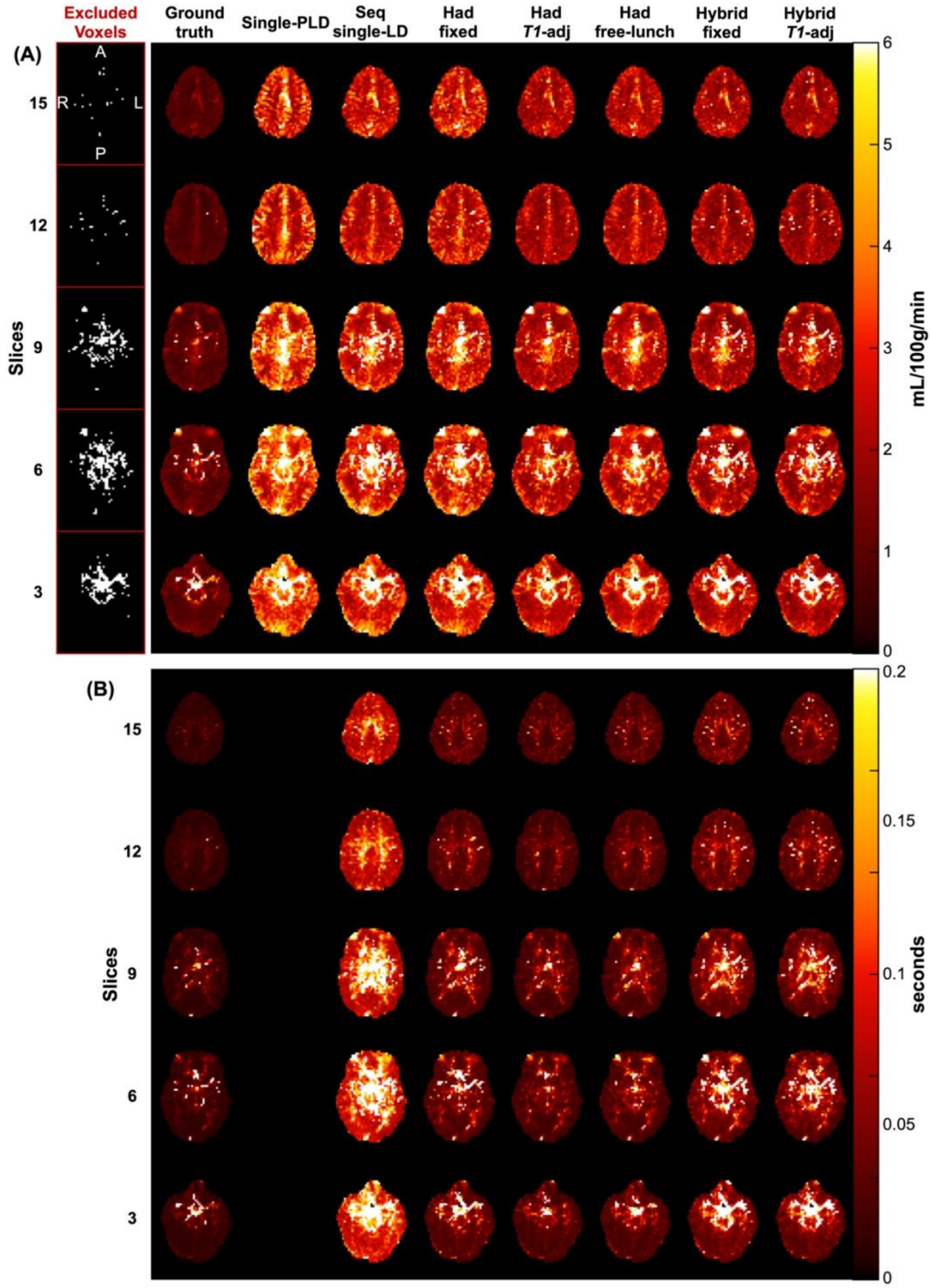

Supporting Information Figure S5: The voxels excluded due to the posterior SD restrictions and the posterior SD maps for 5 slices of a single representative subject. (A) The excluded voxels and the CBF posterior SD maps, (B) the ATT posterior SD maps. The single-PLD protocol does not have ATT posterior SD maps because ATT is not estimated. The excluded voxel maps show voxels excluded due to high SDs in either the CBF or the ATT posterior SD maps.

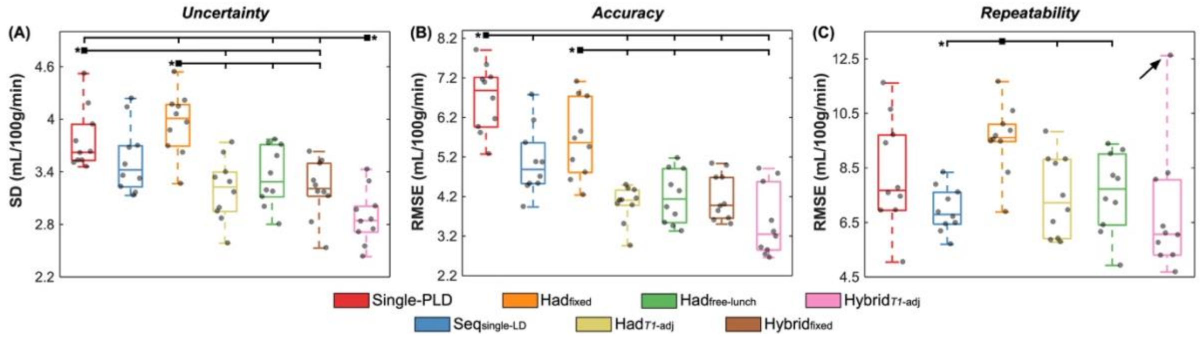

Supporting Information Figure S6: The subjectwise in vivo comparisons of the uncertainty (posterior SDs), accuracy (RMSEs), and repeatability (test-retest RMSEs) metrics for each protocol. The boxplots show the median, quartiles, and range across subjects, in each case. Significant differences are shown for individual protocols (two-sided paired Wilcoxon signed-rank test, Bonferroni correction for 21 comparisons (A, B) and 15 comparison (C), α<0.05). The black arrow in (C) highlights an outlier subject for Hybrid$_{T1\text{-}adj}$; see section 4.7 for details.

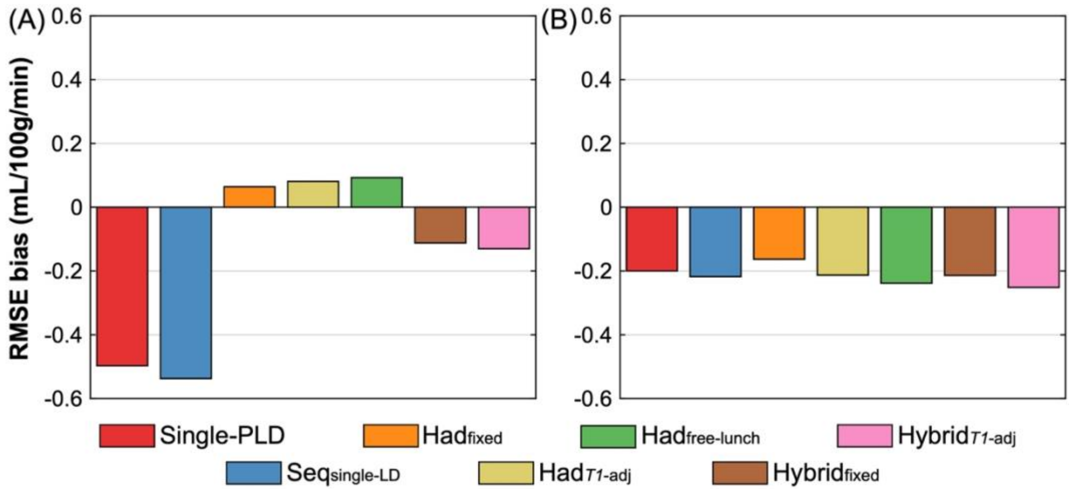

Supporting Information Figure S7: Bias in the ground truth MC simulation CBF accuracy estimates (RMSEs) when using 2 different noise models: (A) bias in the ground truth values fitted using 1 noise magnitude for all of the data and (B) bias in the ground truth values fitted using 3 noise magnitudes (1 each for: non-time-encoded protocols, time-encoded protocols, and the hybrid protocols). When 1 noise magnitude is used in the fitting, there is a large variation in the bias across protocols, but when 3 noise magnitude are used the RMSEs are much more similarly underestimated for all the protocols by -0.21 ± 0.03 mL/100g/min.

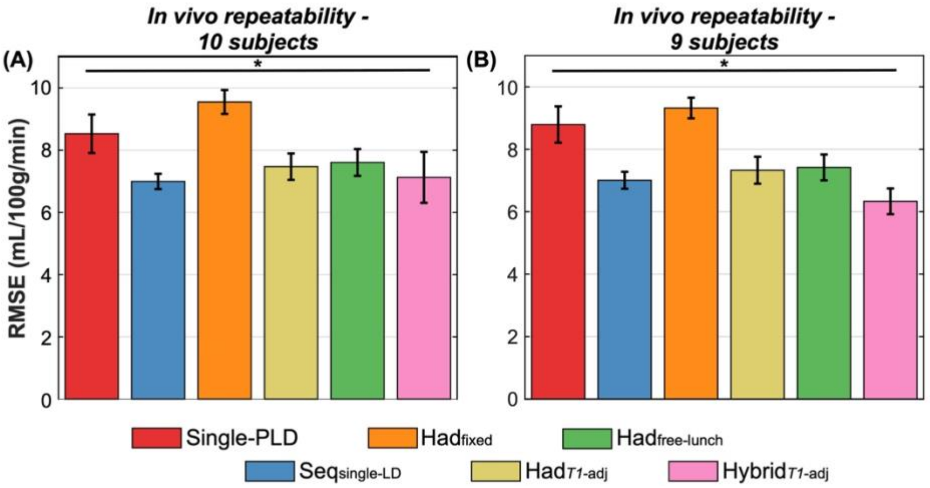

Supporting Information Figure S8: The in vivo repeatability (test-retest RMSEs) across all voxels with all subjects (A) and with 1 subject removed (B). The removed subject had a much larger $Hybrid_{T1\text{-}adj}$ test-retest RMSE than the other subjects, but when removed did not lead to a large change in the test-retest RMSEs of the other protocols. The means and standard errors of the bootstrap distributions are shown (see methods). All differences were significant (two-sided paired Wilcoxon signed-rank test, Bonferroni correction for 15 comparisons, $\alpha<0.05$).

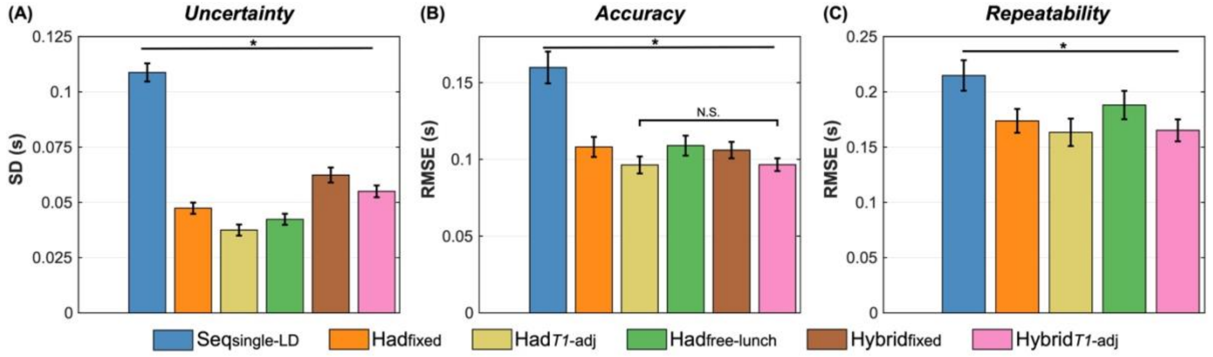

Supporting Information Figure S9: The in vivo voxelwise ATT measures of (A) uncertainty (posterior SDs), (B) accuracy (RMSEs relative to the ground truth estimates) and (C) repeatability (test-retest RMSEs). The mean and standard error (see methods) of the metrics across voxels are shown. All protocols had significantly different measures, unless highlighted as not-significant (N.S.) (two-sided paired Wilcoxon signed-rank test, Bonferroni correction for 15 comparisons (A, B) and 10 comparisons (C), $\alpha<0.05$)..

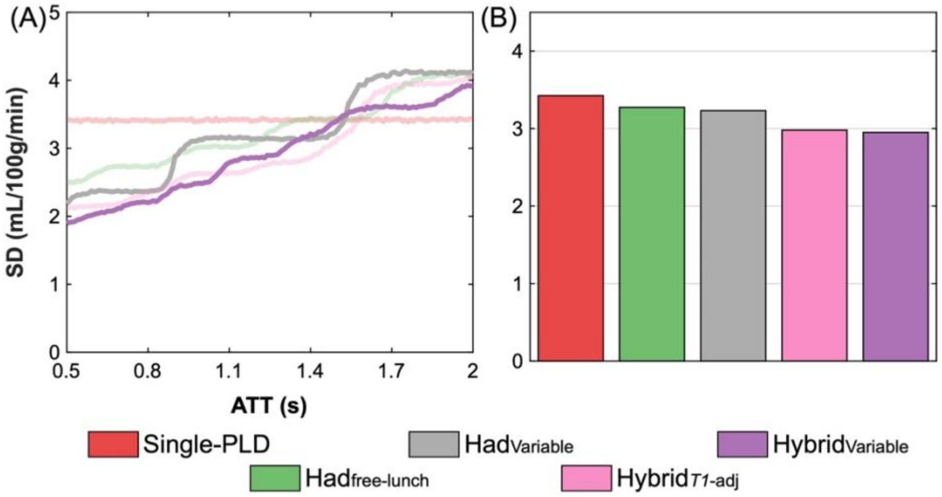

Supporting Information Figure S10: The MC simulation CBF posterior SDs for $Had_{variable}$, $Hybrid_{variable}$, and a selection of previously compared protocols. (A) the median uncertainty for each protocol across ATTs, (B) the mean uncertainty for each protocol across the whole ATT range.

Protocol	***Label durations (ms)***	***Post-label delays (ms)***	***N_T***	***N_{Ave}***	***N_{Acq}***	***Scan duration (min)***
Single-PLD	3125	2000	1	26	52	5:00
$Seq_{single\text{-}LD}$	2800	75, 975, 1600, 2100, 2300, 2300	6	5	60	5:00
$Seq_{multi\text{-}LD}$	2025, 1400, 3425, 4225, 3850	200, 925, 1975, 1475, 2300	5	6	60	5:00
$Had_{free\text{-}lunch}$	3125, 1100, 1100	100	3	12	48	4:51
$Hybrid_{T1\text{-}adj}$	3550, 1050, 625	75, 275, 550, 625	12	3	48	5:00
$Hybrid_{variable}$	3975, 1125, 300<br>3325, 1425, 550<br>3300, 1225, 500<br>3575, 725, 625	200<br>325<br>575<br>675	12	3	48	5:00

Supporting Information Table S2: The optimized protocol timings when the maximum LD was extended to 5 s. For the time-encoded (Had) and hybrid protocols, the LDs are given in chronological order and the number of LDs defines the size of the Hadamard encoding matrix used. For the $Hybrid_{variable}$ protocol, each PLD is associated with the LDs on the same row. N_T is the number of effective PLDs, N_{Ave} is the number of averages, and N_{acq} is the number of acquired volumes for each scan.

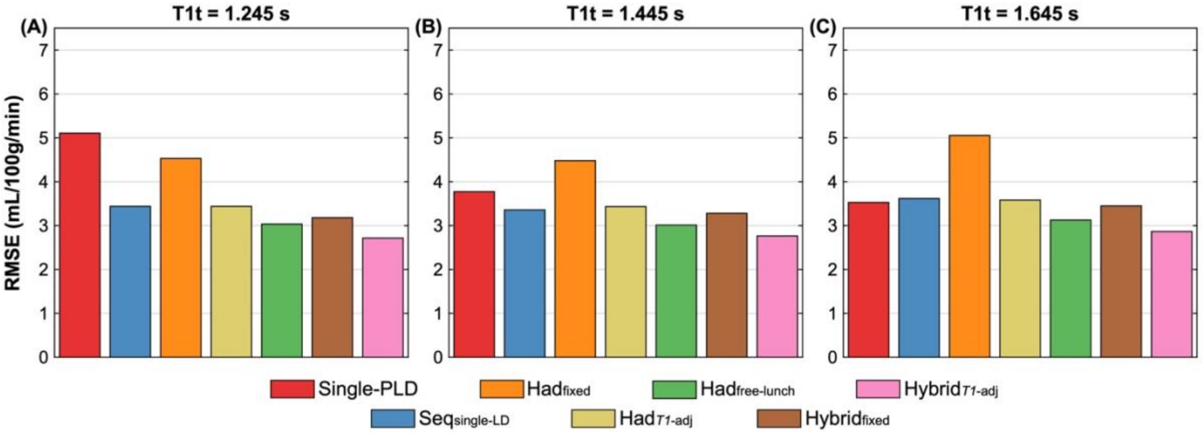

Supporting Information Figure S11: The accuracy of CBF estimates (RMSE relative to ground truth estimates) derived from MC simulations with a uniform ATT distribution from 0.5 to 2 s. The true tissue T_1 (T_{1t}) used to simulate the data has been varied while the T_{1t} used in the fitting was kept constant at 1.445 s. The ground truth estimates are fit in the same way as the individual protocol data, so contain a similar bias. The RMSEs of the protocols, and their relative performances, are similar in each case, except for single-PLD. The single-PLD RMSEs decrease when the true T_{1t} is greater than the value used in fitting and increase when the true T_{1t} is less than the fitting T_{1t}. In all three cases, $Had_{free\text{-}lunch}$ is the best performing literature protocol, while $Hybrid_{T1\text{-}adj}$ is the best performing protocol overall.
